# Histidine re-sensitizes pediatric acute lymphoblastic leukemia to 6-mercaptopurine through tetrahydrofolate consumption and SIRT5-mediated desuccinylation

**DOI:** 10.1038/s41419-024-06599-5

**Published:** 2024-03-14

**Authors:** Na Dong, Hui-Xian Ma, Xue-Qin Liu, Dong Li, Ling-Hong Liu, Qing Shi, Xiu-Li Ju

**Affiliations:** 1 Department of Pediatrics, Qilu Hospital of Shandong University, Jinan, 250012 Shandong Province China; 2 Cryomedicine Laboratory, Qilu Hospital of Shandong University, Jinan, 250012 Shandong Province China

**Keywords:** Paediatric cancer, Acute lymphocytic leukaemia, Cancer therapeutic resistance

## Abstract

Despite progressive improvements in the survival rate of pediatric B-cell lineage acute lymphoblastic leukemia (B-ALL), chemoresistance-induced disease progression and recurrence still occur with poor prognosis, thus highlighting the urgent need to eradicate drug resistance in B-ALL. The 6-mercaptopurine (6-MP) is the backbone of ALL combination chemotherapy, and resistance to it is crucially related to relapse. The present study couples chemoresistance in pediatric B-ALL with histidine metabolism deficiency. Evidence was provided that histidine supplementation significantly shifts the 6-MP dose-response in 6-MP-resistant B-ALL. It is revealed that increased tetrahydrofolate consumption via histidine catabolism partially explains the re-sensitization ability of histidine. More importantly, this work provides fresh insights into that desuccinylation mediated by SIRT5 is an indispensable and synergistic requirement for histidine combination therapy against 6-MP resistance, which is undisclosed previously and demonstrates a rational strategy to ameliorate chemoresistance and protect pediatric patients with B-ALL from disease progression or relapse.

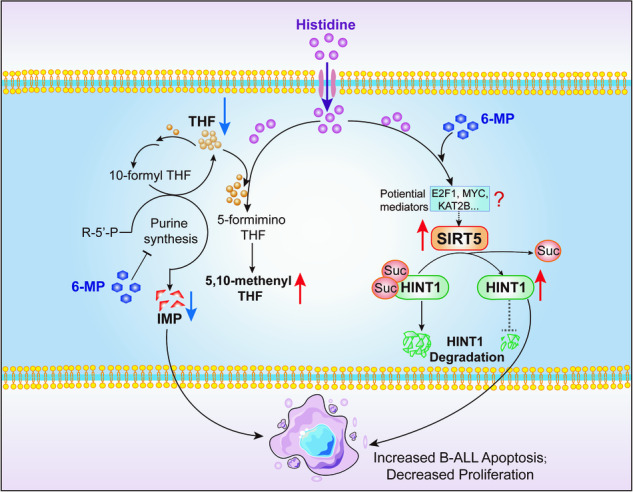

## Introduction

Acute lymphoblastic leukemia (ALL) is the most common malignancy among children, with a global incidence of ~30 cases per million; 85% of the cases are classified as precursor B-cell lineage (B-ALL) [1]. The survival rate of pediatric ALL has dramatically increased to 90%, having benefited from progressive improvements in the efficacy of multiagent chemotherapy regimens and advances in supportive care [2, 3]. However, disease progression and relapse following chemoresistance still occur in ~10–20% of pediatric patients with ALL and it remains the leading cause of mortality among all childhood malignancies. Further efforts are required to ameliorate drug resistance and reduce undesirable clinical outcomes.

Recent studies have identified that an overwhelming majority of pediatric ALL recurrences arise in the consolidation and maintenance therapy phases [4]. The backbone of maintenance therapy is a prolonged daily oral administration of 6-mercaptopurine (6-MP) for approximately two years [2]. Resistance to 6-MP and toxicity-related treatment interruption are closely related to survival time, relapse rate, and second malignant neoplasm incidence [5–8]. Although a growing number of studies are dedicated to identifying the genetic and metabolic mechanisms involved in 6-MP resistance, no effective clinical strategy for overcoming resistance has yet been defined [7, 9, 10].

Extensive preclinical studies have demonstrated that reprogrammed amino acid metabolism in leukemia is responsible for tumor development and altered chemotherapeutic responses [11–14]. Histidine (His) is a nutritionally essential amino acid that shows clear benefits in the treatment of a wide range of conditions [15–18]. Notably, histidine supplementation has recently been reported to increase the utilization of tetrahydrofolate (THF) via the histidine degradation pathway, thus revealing a promising novel strategy to overcome chemoresistance [19, 20].

Recently, succinylation was identified as a novel natural post-translational modification (PTM) of lysine residues [21–25]; however, the underlying mechanisms by which succinylation influences tumor development and drug resistance remain poorly elucidated. Accumulating evidence suggests that sirtuin 5 (SIRT5) acts as a crucial eraser of lysine succinylation with potent metabolic regulatory activities [23, 26]. SIRT5 modulates tumorigenesis and metastasis in a context-specific manner and depends on its deacylation activity [27–30]. However, our knowledge of its biological functions in B-ALL remains limited.

In the present study, we elucidated that defects in histidine metabolism were associated with chemoresistance in pediatric B-ALL. Histidine re-sensitized B-ALL to 6-MP in drug-resistant cells and xenograft mice model. The anti-leukemic capacity of histidine supplementation was attributed to the synergistic action of increased THF utilization via the histidine degradation pathway and SIRT5-mediated desuccinylation of histidine triad nucleotide-binding protein 1 (HINT1). These results provide a rationale for the potential application of histidine to overcome 6-MP resistance in pediatric patients with refractory/relapsed B-ALL.

## Results

### Histidine metabolism is associated with chemoresistance in B-ALL

**Fig. 1:**
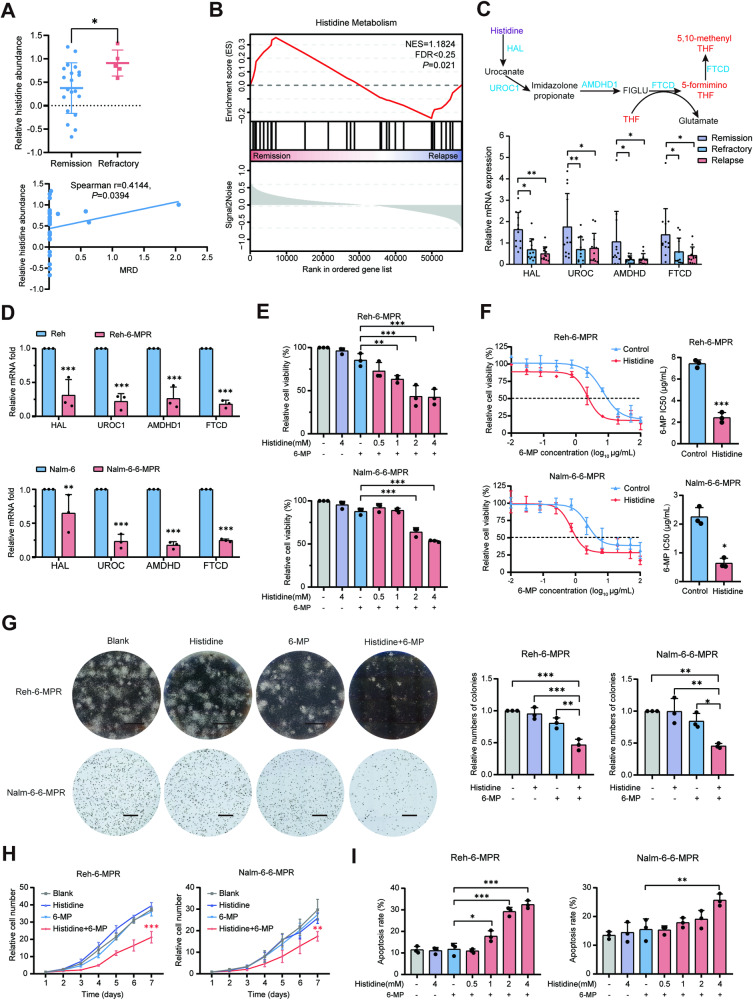
Histidine metabolism is associated with chemoresistance in B-ALL. **A** Relative abundance of histidine in plasma in children with B-ALL at initial diagnosis, comparing remission (*n* = 20) and refractory (*n* = 5) cases (top panel). Correlation between histidine levels and minimal residual disease (MRD) (bottom panel). **B** Gene set enrichment analysis (GSEA) of the gene expression profiles from the TARGET ALL dataset. **C** The mRNA expression of genes involved in histidine catabolism (*HAL*, *UROC*, *AMDHD*, and *FTCD*) among pediatric patients with B-ALL in the remission, refractory, and relapsed groups (*n* = 12). **D** Comparison of mRNA expression levels between Reh-6-MPR, Nalm-6-6-MPR cells, and their respective parental cell lines (*n* = 3). **E** Cell viability of Reh-6-MPR and Nalm-6-6-MPR cells treated with the indicated agents for 48 h, measured by CCK-8 assay. 6-MP was applied at a concentration of 1.0 μg/mL in Reh-6-MPR and 0.5 μg/mL in Nalm-6-6-MPR cells (*n* = 3). **F** Drug sensitivity curves and IC50 values of Reh-6-MPR and Nalm-6-6-MPR cells after treatment with increasing concentrations of 6-MP (0–100 µg/mL) alone or combined with histidine (4 mM) for 48 h (*n* = 3). **G** Representative images and quantification of the colony formation assay in Reh-6-MPR and Nalm-6 6-MPR cells treated with PBS, histidine (4 mM), 6-MP (1.0 μg/mL in Reh-6-MPR and 0.5 μg/mL in Nalm-6-6-MPR cells), or a combination of histidine and 6-MP (*n* = 3). Scale bar = 2 mm. **H** Cell growth curves of Reh-6-MPR and Nalm-6-6-MPR cells treated with the indicated agents (*n* = 3). **I** Apoptotic proportion of Reh-6-MPR and Nalm-6-6-MPR cells treated with the indicated agents for 48 h, measured by flow cytometry with FITC-Annexin V/PI staining (*n* = 3). Data were presented as mean ± SEM. *P < 0.05, **P < 0.01, ***P < 0.005.

Firstly, we performed a liquid chromatography‑mass spectrometry (LC-MS)‑based global metabolomic analysis of the peripheral blood plasma samples from 25 children newly diagnosed with B-ALL. A significantly higher abundance of histidine at initial diagnosis was observed in children with refractory B-ALL compared to those in the remission group. Histidine levels appeared to be positively correlated with measurable residual disease (MRD) [31] after standard induction chemotherapy (Fig. 1A and Supplementary Table 1). Gene set enrichment analysis (GSEA) of the gene expression profiles from the Therapeutically Applicable Research to Generate Effective Treatments (TARGET) ALL dataset (*n* = 207) was performed [32], which revealed that the histidine metabolic pathway was abnormally enriched in patients in remission (*n* = 134) compared to patients with relapse (*n* = 73) (Fig. 1B).

To further substantiate the role of histidine metabolism in the response to B-ALL treatment, the expression levels of the main enzymes involved in histidine catabolism (*HAL*, *UROC*, *AMDHD*, and *FTCD*) were examined. Our clinical samples from pediatric patients with B-ALL showed that these genes were significantly downregulated in the refractory/relapsed group compared to the remission group (Fig. 1C). We subsequently generated 6-MP drug-resistant B-ALL cell lines, namely Reh-6-MPR and Nalm-6-6-MPR (Fig. S1A) and found that the mRNA levels of genes involved in the histidine degradation pathway were downregulated in the drug-resistant group compared to the control groups (Fig. 1D). Consistently, LS-MS analysis revealed slightly higher intracellular histidine levels in 6-MP resistant cells compared to the parental strains (Fig. S1B). Therefore, we concluded that the defective histidine catabolism may contribute to elevated histidine levels and undesirable chemotherapy responses in B-ALL.

A recent study reported that histidine supplementation can upregulate the histidine metabolic pathway [19]. Based on the previous study and our findings, we investigated the biological effects of histidine on 6-MP-resistant B-ALL cells. CCK-8 assay results showed that Reh-6-MPR and Nalm-6-6-MPR cells were resistant to 6-MP at the concentrations tested (1.0 μg/mL in Reh-6-MPR and 0.5 μg/mL in Nalm-6-6-MPR cells); however, when combined with histidine supplementation, 6-MP significantly reduced the viability of Reh-6-MPR and Nalm-6-6-MPR cells (Fig. 1E). In Reh-6-MPR and Nalm-6-6-MPR cells, histidine treatment resulted in a greater than three-fold decrease in the half maximal inhibitory concentration (IC50) of 6-MP compared to that in the controls (Fig. 1F). However, this result was not observed in the corresponding normal cell lines (Fig. S1C), demonstrating that histidine re-sensitized drug-resistant B-ALL cells to 6-MP. No similar difference in the IC50 of cyclophosphamide (CTX) was observed in the histidine-treated CTX-resistant leukemia cells (Fig. S1D). Moreover, colony formation and cell growth assays were employed to further clarify the proliferation-inhibiting effects of histidine. As expected, in the presence of 6-MP, histidine inhibited colony formation and proliferation of 6-MPR B-ALL cells (Fig. 1G, H). Additionally, histidine significantly promoted 6-MP-induced apoptosis and cell cycle arrest at the G0/G1 phase in 6-MPR cells in a dose-dependent manner (Fig. 1I and S1E). Collectively, these results confirmed that histidine significantly shifted the 6-MP dose-response and reversed the chemoresistance of B-ALL cells to 6-MP in vitro.

### Histidine supplementation increases 6-mercaptopurine sensitivity in patients with B-ALL and mouse model

**Fig. 2:**
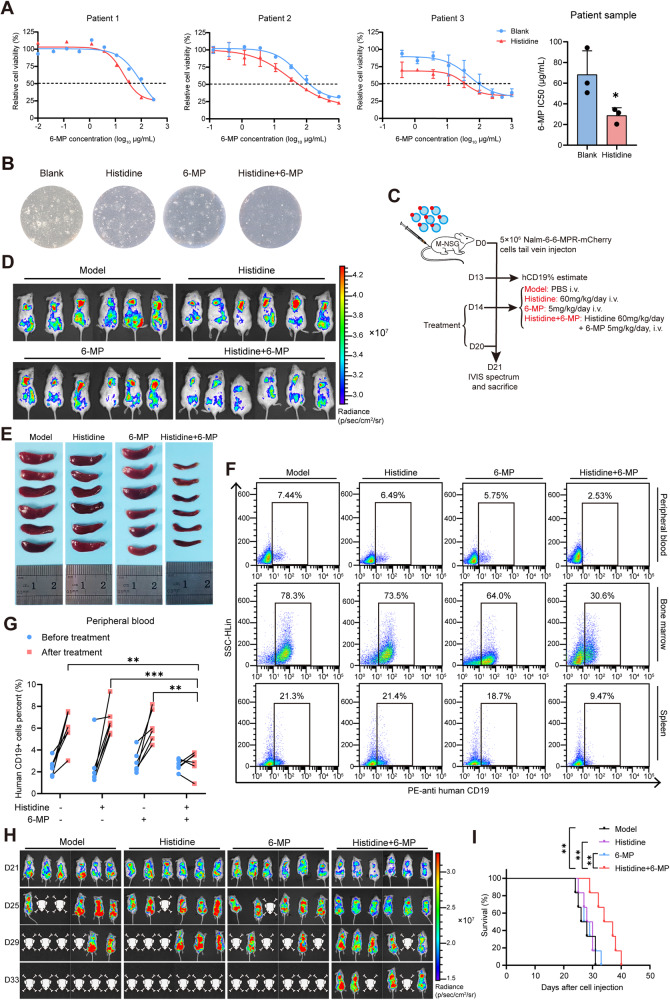
Histidine supplementation increases 6-mercaptopurine sensitivity in B-ALL patients and mouse model. **A** Drug sensitivity curves and IC50 values of bone marrow mononuclear cells derived from pediatric patients with relapsed B-ALL (*n* = 3). **B** Representative images of the colony formation assay in primary B-ALL cells treated with PBS, histidine, 6-MP, or a combination of histidine and 6-MP. **C** Schematic diagram depicting the in vivo assessment of histidine supplementation for rescuing sensitivity to 6-MP. **D** Representative living images of mice engrafted with Nalm-6-6-MPR cells acquired by detecting the mCherry signal via IVIS spectrum at the endpoint of treatment (*n* = 6). **E** Images of spleens isolated from mice at the endpoint of treatment (*n* = 6). **F** Representative flow cytometry images of the percentage of human CD19^+^ B-ALL cells in peripheral blood, bone marrow, and spleen at the endpoint of indicated treatment. **G** Quantification of human CD19^+^ cells in peripheral blood of mice before (D14) and after (D21) the indicated treatment (*n* = 6). **H** In vivo leukemic burden monitoring images of NSG mice engrafted with Nalm-6-6-MPR cells post-treatment. **I** Kaplan–Meier survival curves of NSG mice in the indicated groups (*n* = 6). Data were presented as mean ± SEM. *P < 0.05, **P < 0.01, ***P < 0.005.

Furthermore, to confirm the generalizability of our observations, primary leukemia cells from the bone marrow were isolated from relapsed pediatric patients with B-ALL and then treated with histidine and 6-MP in the same manner as for the 6-MPR cell lines. Consistent with the previous results, histidine significantly rescued the sensitivity to 6-MP, resulting in a further decrease in cell viability (Fig. 2A) and colony-forming capacity (Fig. 2B and S2A).

To determine whether histidine re-sensitized B-ALL to 6-MP in vivo, the xenotransplantation mouse model was established by engrafting M-NSG mice with Nalm-6-6-MPR cells (Fig. S2B–E). As shown in the procedure diagram (Fig. 2C), mice bearing B-ALL were treated with vehicle, histidine, 6-MP, or combination therapy (histidine and 6-MP) intravenously for 7 days. The 6-MP-resistant B-ALL mice treated with 6-MP alone did not show a significant decrease in leukemia load. However, the combined histidine and 6-MP treatment resulted in a significant decrease in tumor burden, as monitored by the in vivo imaging system (IVIS) (Fig. 2D and S2F). Additionally, splenomegaly caused by the infiltration of leukemia cells was further attenuated in the combined treatment group (Fig. 2E and S2G). The frequencies of human CD19^+^ B-ALL cells in the peripheral blood, bone marrow, and spleen were reduced in the combined treatment group compared to the vehicle or monotherapy groups (Fig. 2F, G and S2H, I). We further conducted in vivo leukemic burden monitoring using IVIS on an additional group of NSG mice after treatment as described above. Consistently, the combination of histidine and 6-MP intervention significantly delayed leukemic progression and extended survival time (Fig. 2H, I). Taken together, these results reveal that histidine supplementation, in combination with chemotherapeutic agents, represents a rational strategy to improve the outcomes in drug-resistant B-ALL.

### Tetrahydrofolate (THF) consumption via the histidine degradation pathway accounts for the efficacy of histidine in reverse chemoresistance

**Fig. 3:**
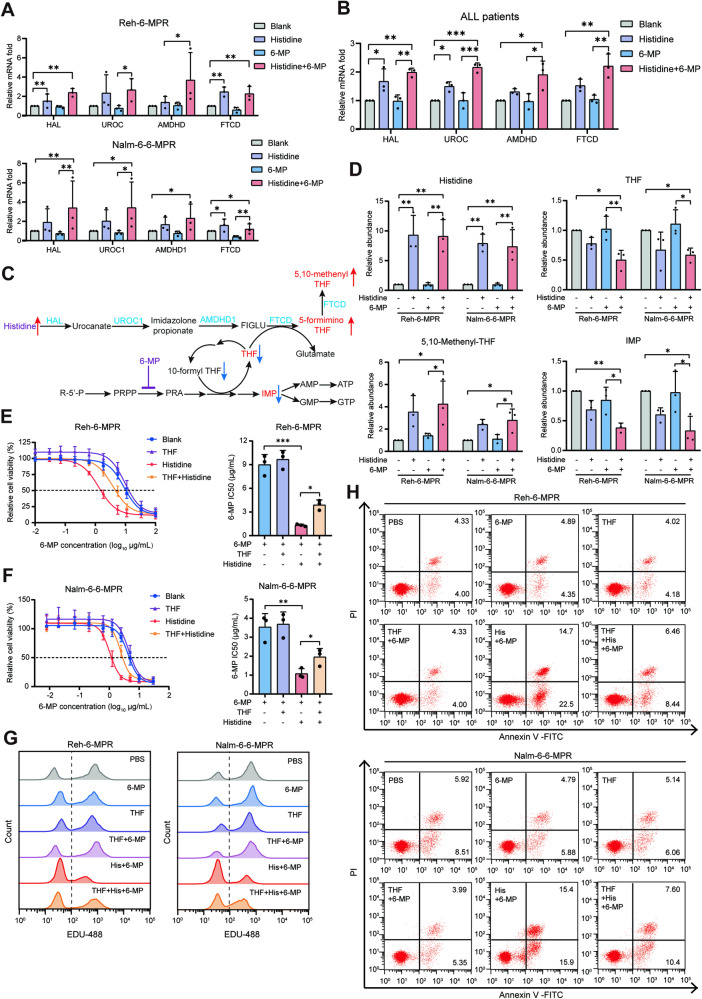
Tetrahydrofolate (THF) consumption through the histidine degradation pathway accounts for the efficacy of histidine in reversing 6-MP resistance. **A** Comparison of the mRNA expression of *HAL*, *UROC*, *AMDHD*, and *FTCD* genes in Reh-6-MPR and Nalm-6-6-MPR cells treated with PBS, histidine, 6-MP, or a combination of histidine and 6-MP (*n* = 3). **B** The mRNA expression of key enzymes involved in histidine catabolism among pediatric patients with relapsed B-ALL treated with histidine, alone or combined with 6-MP (*n* = 3). **C** Schematic of histidine degradation and de novo purine synthesis pathway, and their potential interaction. **D** Intracellular levels of metabolites measured by LS-MS in Reh-6-MPR and Nalm-6-6-MPR cells cultured under the indicated conditions (*n* = 3). **E**, **F** Drug sensitivity curves and corresponding IC50 values of Reh-6-MPR cells (**E**) and Nalm-6-6-MPR cells (**F**) after treatment with increasing concentrations of 6-MP, in the presence of PBS, THF (7.5 μM), histidine, or a combination of histidine and THF for 48 hours (*n* = 3). **G**, **H** Representative flow cytometric images of EdU proliferation assay (**G**) and the apoptotic assay (**H**). Data were presented as mean ± SEM. *P < 0.05, **P < 0.01, ***P < 0.005.

To validate the activation of the histidine degradation pathway induced by histidine supplementation, real-time quantitative PCR (RT-qPCR) was used to evaluate the mRNA expression of key enzymes in the pathway. The result yielded that histidine supplementation significantly evoked its catabolism in 6-MPR cells (Fig. 3A) and primary samples from patients with relapsed ALL (Fig. 3B). To provide insights into the subsequent metabolic changes caused by histidine supplementation, we measured the levels of the main metabolites in the histidine degradation and de novo purine synthesis pathways (Fig. 3C). LC-MS analysis demonstrated an ~5- to 11-fold increase in intracellular histidine levels after histidine treatment. We observed a statistically significant decrease in THF levels, a critical cofactor in the de novo nucleotide synthesis pathway, and an increase in the downstream metabolite 5,10-methenyl THF in histidine-treated cells. Additionally, in the presence of 6-MP, histidine treatment resulted in markedly lower levels of imidazole propionate (IMP) compared to other treatment groups (Fig. 3D), without alterations in the abundance of intracellular 6-MP (Fig. S3A).

We further treated Reh-6-MPR and Nalm-6-6-MPR cells with exogenous THF to maintain intracellular THF pools in the presence of histidine and 6-MP. When treated with THF, the IC50 of 6-MP in histidine-treated cells showed a significant increase, suggesting that THF partially offset the antitumor effects of the combined treatment with 6-MP and histidine (Fig. 3E, F). Consistent with this result, after THF supplementation, the suppressed cell proliferation, elevated apoptotic frequency, and cell cycle arrest exhibited by 6-MPR cells treated with both 6-MP and histidine were significantly restored towards the state of the control groups (Fig. 3G, H and S3B–D). Collectively, these data indicate that exhaustion of the THF pool via the histidine degradation pathway partially contributed to the increased 6-MP-mediated inhibition of purine synthesis and leukemic cell growth observed in histidine-treated cells.

### SIRT5 plays a crucial role in the anti-leukemic effect induced by the combination of 6-MP and histidine

**Fig. 4:**
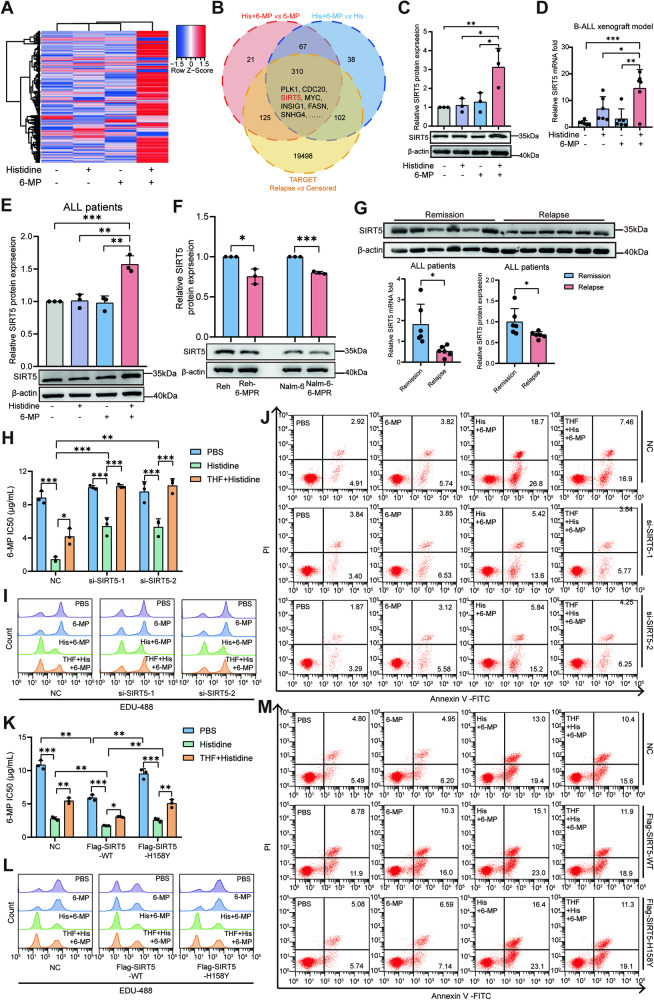
SIRT5 plays a crucial role in the anti-leukemic effect induced by the combination of 6-MP and histidine. **A** Heatmap showing differentially regulated genes identified by RNA-seq in the Reh-6-MPR cell line after treatment with PBS, histidine, 6-MP, or both histidine and 6-MP. **B** Venn diagram illustrating the intersection of differentially expressed genes. **C**–**E** Validation of upregulated SIRT5 expression after treatment with both histidine and 6-MP in Nalm-6-6-MPR cells (**C**; *n* = 3), and bone marrow-derived from xenotransplantation mice (**D**; *n* = 6) and primary B-ALL samples (**E**; *n* = 3). **F** Comparison of SIRT5 protein expression between 6-MP resistant leukemia cell lines and their relevant parental strains (*n* = 3). **G** The mRNA and protein levels of SIRT5 in the relapse and remission primary B-ALL cohorts (*n* = 6). **H** Comparison of the IC50 of 6-MP in *SIRT5*-downregulated and control leukemia cells after treatment with increasing concentrations of 6-MP, in the presence of PBS, histidine, or a combination of THF and histidine for 48 h (*n* = 3). **I**, **J** Representative flow cytometric images of EdU proliferation assay (**I**) and apoptotic assay (**J**) in SIRT5-downregulated and control leukemia cells treated with the indicated agents for 48 h (*n* = 3). **K** Comparison of the IC50 of 6-MP in SIRT5-WT/ SIRT5-H158Y-overexpressed and control leukemia cells (*n* = 3). **L**, **M** Representative flow cytometric images of EdU proliferation assay (**L**) and apoptotic assay (**M**) in SIRT5-WT/SIRT5-H158Y-overexpressed and control leukemia cells treated with the indicated agents for 48 h. Data were presented as mean ± SEM. *P < 0.05, **P < 0.01, ***P < 0.005.

However, based on the above results, we observed that THF was not sufficient to completely alter histidine-induced re-sensitization to 6-MP, which could indicate the possible participation of other elements. To identify genes that contribute to the response of leukemia cells to 6-MP and histidine, we performed RNA-seq of Reh-6-MPR cells after treatment with vehicle, histidine, 6-MP, or combined histidine and 6-MP. The heatmap demonstrated a different expression pattern in the combined treatment compared to the other groups (Fig. 4A). Subsequently, differentially expressed genes (DEGs) between the combined treatment group and monotherapy groups (histidine or 6-MP clone) in our RNA-seq data, along with DEGs between the relapsed and censored groups in the TARGET ALL dataset [32], were integratedly analyzed employing Venn diagram analysis (Fig. 4B). Among the DEGs, we identified SIRT5 from the intersection in the Venn diagram and validated that the mRNA and protein expression levels of SIRT5 in the cell lines were significantly increased when 6-MP was combined with histidine compared to other treatment groups (Fig. 4C and S4A). This result was further confirmed by identifying an increase in SIRT5 levels in the bone marrow-derived from xenografted mice (Fig. 4D) and patients with primary B-ALL (Fig. 4E and S4B) in the combined treatment group compared to other groups. Meanwhile, we identified a reduction in SIRT5 expression in Reh-6-MPR and Nalm-6-6-MPR cell lines, compared to their relevant parental strains (Fig. 4F and S4C). Consistently, primary samples obtained from pediatric patients with B-ALL demonstrated that SIRT5 was significantly downregulated in the relapsed group compared to the remission group (Fig. 4G). Notably, SIRT5 induction was also observed in normal 6-MP sensitive cell lines (Reh and Nalm-6) under the combination of histidine and 6-MP (Fig. S4D, E), although the drug sensitivity remained unaffected (Fig. S1C). These findings suggest that SIRT5 may play a crucial role in the re-sensitization efficiency of histidine, particularly in drug-resistant leukemia cells.

To further substantiate the role of SIRT5 in the suppressive effect of the combined histidine and 6-MP treatment, two siRNAs targeting *SIRT5* were used to downregulate its expression, as verified by RT-qPCR and western blotting (Fig. S4F). *SIRT5* knockdown significantly increased the IC50 of 6-MP in the presence of histidine (Fig. 4H and S4G). Knockdown of SIRT5 alone significantly decreased the sensitivity of normal Reh and Nalm-6 cell lines to 6-MP, even in the absence of histidine supplementation (Fig. S4H). In addition, *SIRT5* knockdown re-activated cell proliferation, as demonstrated in EdU assays (Fig. 4I and S4I), and decreased the apoptosis induced by histidine and 6-MP (Fig. 4J and S4J). Notably, the addition of THF to the SIRT5-inhibited leukemia cells reversed the effects of combined histidine and 6-MP treatment on cellular proliferation and apoptosis to levels similar to the control treatment group.

SIRT5 is an important tumor regulator because of its potent deacetylase, desuccinylase, deglutarylase, and demalonylase activities [23, 26, 33, 34]. To test whether the effects on leukemia cells induced by SIRT5 were mediated by its enzymatic activity, cell lines overexpressing wild-type SIRT5 (SIRT5-WT) and SIRT5-H158Y, a catalytically inactive mutant of SIRT5 [28, 29, 35], were established using specific plasmids (Fig. S4K). The ectopic expression of SIRT5 partially mimicked the anti-leukemic effect of histidine combined with 6-MP. Specifically, SIRT5 overexpression reduced the IC50 of 6-MP in 6-MPR B-ALL cells (Fig. 4K and S4L), suppressed cell proliferation (Fig. 4L and S4M), and promoted 6-MP-induced apoptosis (Fig. 4M and S4N). However, the overexpression of SIRT5-H158Y resulted in no significant changes in cellular proliferation and apoptosis. Collectively, these results announced that upregulation of SIRT5 combined with THF utilization accounts for the inhibitory effects of the combined 6-MP and histidine treatment on leukemia cells, and that enzymatic activity is essential for SIRT5 to be effective.

### SIRT5-mediated desuccinylation of HINT1 impairs cell viability in drug-resistant B-ALL

**Fig. 5:**
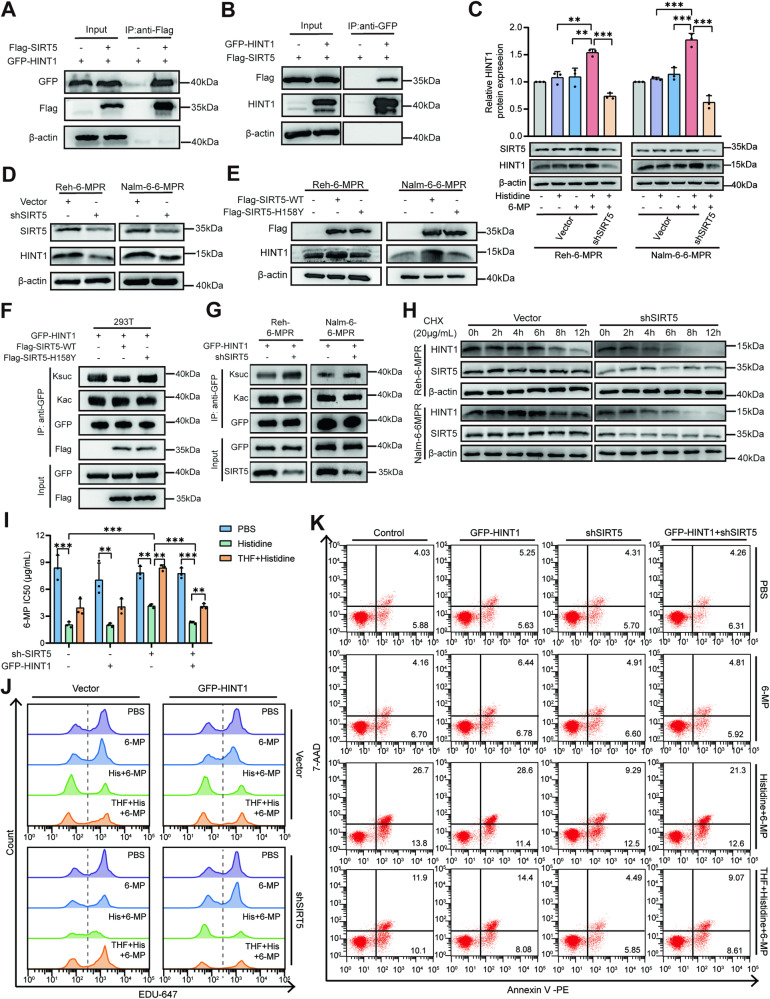
SIRT5-mediated desuccinylation of HINT1 impairs cell viability in drug-resistant leukemia. **A**, **B** Immunoprecipitation of Flag-SIRT5 or GFP-HINT1 using anti-Flag (**A**) or anti-GFP (**B**) antibody-conjugated beads, respectively, and detection of the interaction between HINT1 and SIRT5 by immunoblotting with the indicated antibodies. **C** HINT1 protein expression in Reh-6-MPR and Nalm-6-6-MPR cells treated with histidine, alone or combined with 6-MP (*n* = 3). **D**, **E** Examination of HINT1 protein expression by western blotting in Reh-6-MPR and Nalm-6-6-MPR cells with SIRT5 knocked down using lentivirus (**D**) or SIRT5 overexpressed by plasmid transfection (**E**). **F** Co-expression of GFP-HINT1 with SIRT5-WT or SIRT5-H158Y in 293 T cells, and determination of acetylation and succinylation levels of GFP-bead-immunoprecipitated HINT1 by immunoblotting with pan-acetyl-K and pan-succinyl-K antibodies. **G** Overexpression of GFP-HINT1 in SIRT5-knockdown Reh-6-MPR and Nalm-6-6-MPR cells, and examination of acetylation and succinylation levels of GFP-bead-immunoprecipitated HINT1. **H** Assessment of HINT1 half-lives by western blotting in Reh-6-MPR and Nalm-6-6-MPR cells treated with cycloheximide (CHX, 20 mg/mL) for 0, 2, 4, 6, 8, 12 h. **I** Comparison of the IC50 of 6-MP in HINT-overexpressed and/or SIRT5-knocked-down leukemia cell (*n* = 3). **J**, **K** Representative flow cytometric images of EdU proliferation assay (**J**) and apoptotic assay (**K**) in HINT-overexpressed and/or SIRT5-knocked-down leukemia cells. Data are presented as mean ± SEM. *P < 0.05, **P < 0.01, ***P < 0.005. Ksuc succinyllysine, Kac acetyllysine.

To further identify the downstream targets of SIRT5, Flag-tagged SIRT5 was overexpressed in Nalm-6-6-MPR cells and immunoprecipitated using Flag-trap agarose. Co-precipitated proteins were eluted and identified by tandem mass spectrometry. The interaction between HINT1 and SIRT5 was identified and further confirmed by co-immunoprecipitation assays. Ectopically expressed Flag-SIRT5 pulled down ectopically expressed GFP-HINT1 in 293 T cells (Fig. 5A), and could be co-immunoprecipitated with the GFP-HINT1 reversely (Fig. 5B). HINT1 is regarded as a tumor suppressor in various malignancies [36, 37]. The combination of histidine and 6-MP effectively upregulated HINT1 protein expression (Fig. 5C), without altering the transcription level of HINT1 (Fig. S5A). Lentiviral SIRT5-knockdown led to a significant reduction in the HINT1 protein level in Reh-6-MPR and Nalm-6-6-MPR cells (Fig. 5C, D and S5B), while leaving the corresponding mRNA levels unaffected (Fig. S5C). Consistently, *SIRT5*-overexpression increased the relative abundance of HINT1 protein (Fig. 5E and S5D). These findings suggest that the mechanism responsible for the increased HINT1 protein is independent of transcriptional regulation.

Given the well-defined function of SIRT5 in PTM, we next investigated which enzymatic activity acts on the target protein HINT1, using pan-acetyl-K and pan-succinyl-K antibodies [35, 38]. The acetylation and succinylation levels of GFP-bead-immunoprecipitated HINT1 were determined using western blot analysis in GFP-HINT1-overexpressed 293T, Reh-6-MPR, and Nalm-6-6-MPR cells. Overexpression of SIRT5-WT, but not SIRT5-H158Y, caused a significant reduction in the succinylation of HINT1 (Fig. 5F and S5E). Additionally, *SIRT5*-knockdown cells exhibited greater HINT1 succinylation, whereas HINT1 acetylation remained largely unaffected (Fig. 5G and S5F). Owing to the larger size of the succinyl group compared to other typical modification groups, desuccinylation may influence protein structure and stability to a greater extent than other modifications [39, 40]. To determine whether the protein abundance of HINT1 affected by SIRT5 was mediated by desuccinylation-related variations in HINT1 stability, cells were treated with cycloheximide (CHX), a protein synthase inhibitor, and the HINT1 turnover rate was assessed by western blotting. The results showed that *SIRT5* knockdown significantly shortened the half-life of HINT1 (Fig. 5H and S5G).

Furthermore, HINT1 overexpression re-sensitized *SIRT5* knocked down leukemia cells to 6-MP in the presence of histidine (Fig. 5I and S5H). Consistently, HINT1-overexpressed cells showed reduced proliferation (Fig. 5J and S5I) and increased apoptosis (Fig. 5K and S5J). Taken together, these results demonstrated that SIRT5 directly interacts to desuccinylate HINT1 and exerts anti-leukemic effects.

### Knockdown of SIRT5 and THF supplementation significantly impairs the chemo-sensitization efficacy of histidine in vivo

**Fig. 6:**
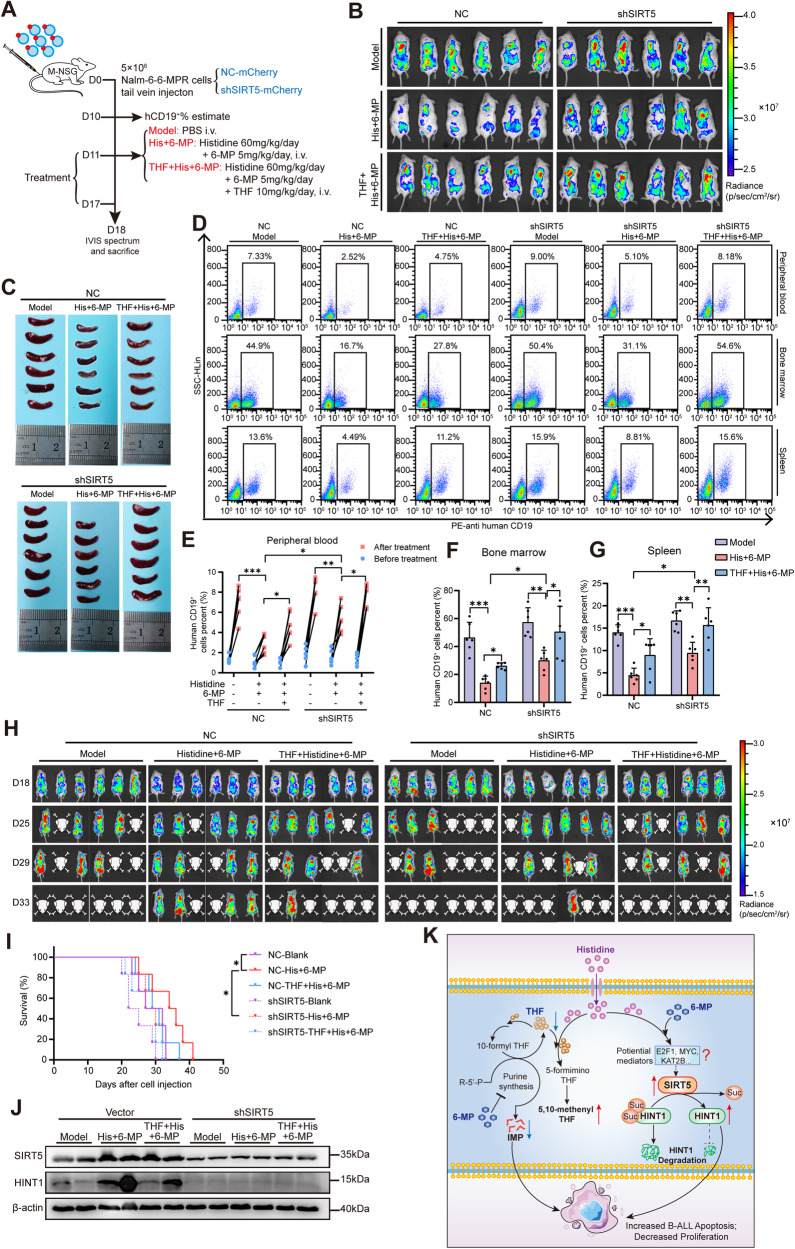
Knockdown of SIRT5 and THF supplementation significantly impairs the chemo-sensitization efficacy of histidine in vivo. **A** Schematic diagram illustrating the in vivo assessment of the role of SIRT5 in leukemia progression in human B-ALL xenografts. **B** Representative living images of mice engrafted with SIRT5-knockdown or control Nalm-6-6-MPR cells acquired through IVIS at the endpoint of treatment (*n* = 6). **C** Images of spleens isolated from mice bearing SIRT5-knockdown or control cells at the endpoint of treatment. **D** Representative flow cytometry images of the percentage of human CD19^+^ B-ALL cells in peripheral blood, bone marrow, and spleen at the endpoint of indicated treatment. **E** Quantification of human CD19^+^ cells in peripheral blood of mice bearing SIRT5-knockdown or control cells before (D10) and after (D18) the indicated treatment (*n* = 6). **F**, **G** Statistical results of human CD19^+^ cells measured by flow cytometry in bone marrow (**F**) and spleen (**G**) of mice at the endpoint of indicated treatment (*n* = 6). **H** In vivo leukemic burden monitoring images of NSG mice engrafted with SIRT5-knockdown or control Nalm-6-6-MPR cells post-treatment with PBS, histidine+ 6-MP, or THF+ histidine+ 6-MP (*n* = 6). **I** Kaplan–Meier survival curves of NSG mice in the indicated groups (*n* = 6). **J** Western blot analysis of SIRT5 and HINT1 in bone marrow-derived from mice bearing SIRT5-knockdown or control Nalm-6-6-MPR cells. **K** Proposed model illustrating the re-sensitization of B-ALL to 6-MP by histidine supplementation through THF consumption and SIRT5-mediated desuccinylation. Data were presented as mean ± SEM. *P < 0.05, **P < 0.01, ***P < 0.005.

To further address the role of SIRT5 in leukemia progression in human B-ALL xenografts, stable *SIRT5*-knockdown or control Nalm-6-6-MPR cell lines were transplanted into M-NSG mice, and mouse leukemia progression was monitored (Fig. 6A). Analysis of the IVIS spectrum revealed a trend toward a greater leukemia load in the SIRT5-knockdown group than in control mice. SIRT5 suppression significantly reduced the antitumor efficacy of the combined treatment (histidine and 6-MP), and the resistance was further enhanced with the addition of THF (Fig. 6B and S6A). Mice bearing SIRT5-knockdown cells manifested increased splenomegaly after histidine and 6-MP treatment compared with the control group (Fig. 6C and S6B). Consistent with previous results, flow cytometry confirmed that *SIRT5* inhibition significantly counteracted histidine and 6-MP-mediated disease control and enhanced the human CD19^+^ leukemia frequencies in the peripheral blood, bone marrow, and spleen. More importantly, when THF was simultaneously supplemented in *SIRT5*-knockdown mice, the anti-leukemic efficacy of the histidine and 6-MP combined treatment was completely offset (Fig. 6D–G). Moreover, as demonstrated in Fig. 6H and I, the combination therapy of histidine and 6-MP induced effective suppression of leukemia and conferred a significant survival advantage. However, this advantage was nullified by SIRT5 inhibition and THF supplementation, either alone or in combination. Additionally, we observed suppression of HINT1 in the bone marrow-derived from mice bearing *SIRT5*-knockdown Nalm-6-6-MPR cells (Fig. 6J and S6C, D). Collectively, these observations further confirmed that SIRT5 and THF play crucial synergistic roles in histidine-induced re-sensitization to 6-MP.

## Discussion

Despite the improved survival rates, concerted efforts focused on the small subsets of patients with drug-resistant leukemia are required to modify therapies to circumvent relapse. Based on public databases and our pediatric B-ALL samples, we associated chemoresistance in B-ALL with histidine metabolism deficiencies. Our experimental results provide evidence that histidine supplementation decreases intracellular THF and significantly shifts the 6-MP dose-response in 6-MP-resistant B-ALL cells and the xenograft mice. More importantly, we freshly demonstrated that desuccinylation of HINT1, which is mediated by elevated SIRT5, is vital for the elevated therapeutic efficacy induced by histidine and 6-MP. Collectively, these results suggest that simple dietary histidine supplementation may be a potential strategy for re-sensitizing drug-resistant pediatric patients with B-ALL to 6-MP (Fig. 6K).

6-MP is an essential antimetabolic agent in ALL combination therapy, with prolonged application during the consolidation, intensification, and maintenance phases [2]. Interestingly, these phases are exactly the stages where most pediatric ALL relapses occur [4]. Emerging evidence in ALL settings suggests that 6-MP resistance and drug treatment interruption contribute to an increased risk of relapse and secondary malignant neoplasm incidence [6, 8]. The crucial role of 6-MP therapy in the treatment and recurrence of ALL emphasizes the urgent need to explore the mechanism and corresponding treatment for 6-MP resistance.

Tumor cells are characterized by metabolic reprogramming to fulfill the biosynthetic demands associated with their uncontrolled proliferation, invasion, metastasis, and recurrence [41–43]. The reprogrammed metabolism of specific amino acids has recently been shown to be essential for leukemogenesis and chemotherapy responses in multiple hematologic malignancies [11, 12, 44]. These metabolic vulnerabilities have been recognized as potential therapeutic targets. Kanarek et al. revealed that the depletion of multiple genes in the histidine catabolism pathway significantly decreased the sensitivity of hematopoietic cells to methotrexate [19]. Consistent with these reports, our present study found aberrantly elevated histidine abundance in children with refractory B-ALL. Based on gene expression profiles from the TARGET dataset and our clinical samples, we observed abnormally downregulated histidine metabolism in patients with refractory and relapsed ALL. We demonstrated that the impaired histidine degradation pathway contributes to abnormal histidine levels, resistance to therapeutic agents, and relapse in pediatric B-ALL.

Histidine is an essential amino acid with unique biochemical and physiological properties, such as proton buffering [45], metal ion chelation [46], and antioxidant activity [47], providing a basis for its promising therapeutic potential under a wide range of conditions. THF is well-known as a critical cofactor in the de novo nucleotide synthesis pathway. Histidine catabolism is one of the few pathways that utilize the cellular pool of THF and attracts attention as an enhancer of anti-cancer metabolic therapy [48, 49]. Previous studies have linked the activation of de novo purine biosynthesis with 6-MP resistance [7, 50]. In the current study, we demonstrated that histidine supplementation re-sensitized B-ALL cells to 6-MP both in vitro and in vivo. Our LC-MS analysis suggested that histidine supplementation significantly drains the intracellular THF pool via its degradation pathway. The lower THF availability perturbs the mutual conversion between 10-formyl THF and THF, compromising the capacity of fast-proliferating cells to synthesize nucleotides and, therefore, partly contributing to the role of histidine as a 6-MP therapy enhancer.

Notably, THF supplementation did not completely abolish the anti-leukemic effect of the combined histidine and 6-MP treatment. Subsequently, RNA-seq analysis was performed, and upregulated SIRT5 caught our attention. SIRT5 has been proven to be involved in tumorigenesis and drug resistance in many cancers [28, 30, 51–53], but opposite tumor-suppressor roles have also been found in various tumor treatment models, including liver cancer [54], Kras-induced pancreatic cancer [30], renal cell carcinoma [27], and acute promyelocytic leukemia [55]. Despite an emerging understanding of the role of SIRT5 in diverse malignancies, its underlying function and mechanism in B-ALL remain largely unknown. The results of this study demonstrated that *SIRT5* inhibition significantly impaired the therapeutic response of 6-MP-resistant B-ALL cells to combined treatment with histidine and 6-MP in vitro. Ectopically expressed *SIRT5* partially mimicked the therapeutic effect of histidine therapy, implying a tumor-suppressing capacity of SIRT5 in B-ALL. In addition, leukemia-bearing mice subjected to SIRT5-knockdown exhibited significantly aggravated tumor burden even when treated with histidine and 6-MP. Moreover, it is crucial to note that the introduction of THF in SIRT5-knockdown mice completely nullified the therapeutic effectiveness of the histidine and 6-MP combination treatment against leukemia. These findings collectively reinforce the significance of SIRT5 and THF as vital collaborative facilitators in the process of histidine-induced 6-MP re-sensitization.

To date, SIRT5 has been identified as an eraser of lysine succinylation [23, 56], which has been recently recognized as a reversible PTM process that is extensively involved in many vital cellular processes and plays an important role in various diseases, including malignancies [39]. However, the physiological and pathological roles of succinylation in B-ALL remain largely unknown. Our study demonstrated that SIRT5 interacted directly with the downstream target HINT1, thereby causing desuccinylation modification and increased stability of the HINT1 protein. Many studies have suggested that HINT1 inhibits tumor initiation and progression through transcriptional regulation [36, 37]. This study showed that in the presence of histidine and 6-MP, HINT1 overexpression resulted in a significant reduction in the proliferation induced by *SIRT5*-inhibition in B-ALL cells. Collectively, we provide new insights into how HINT1 desuccinylation mediated by SIRT5 upregulation contributes to the sensitization effect of histidine on 6-MP in B-ALL and the synergistic involvement of THF utilization.

In conclusion, our results illustrate a novel function of histidine in reversing the chemoresistance of B-ALL cells to 6-MP. Furthermore, THF utilization via the histidine degradation pathway and SIRT5-mediated desuccinylation of HINT1 were crucial for histidine combination therapy to ameliorate chemoresistance in B-ALL cells.

Limitations still existed in this study. As mentioned above, the combination group of histidine and 6-MP exhibited significant differences in gene expression patterns compared to the monotherapy groups (Fig. 4A). This implies the potential involvement of upstream mediators and cooperators of SIRT5 in the re-sensitization efficacy of histidine. Transcriptome data revealed that potential transcription factors of SIRT5, such as *MAX*, *E2F1*, *SP1*, *FOXL1*, and *E2F6* (http://jaspar.genereg.net/), were significantly upregulated in the combination group. Moreover, several other differentially expressed genes, like *KAT2B* and *MYC*, were also predicted to cooperate with SIRT5 [57, 58]. However, the precise mechanisms require further exploration, which will be undertaken in our future research. Nevertheless, our results are sufficient to identify histidine supplementation and SIRT5-HINT1 axis activators as potential strategies for eradicating chemo-resistant B-ALL cells. This approach may help prevent disease progression or recurrence and could benefit pediatric patients with B-ALL.

## Materials and methods

### Cell culture

The human B-ALL cell lines Reh and Nalm-6 were obtained from Procell Life Science& Technology Co., Ltd (Wuhan, China) and cultured in 1640 RPMI medium (11875093, Gibco, Carlsbad, CA, USA) supplemented with 10% fetal bovine serum (FBS, A3161002C, Gibco) and 1% penicillin/streptomycin (C100C5, New Cell & Molecular Biotech, Suzhou, China). The 293T cell line was maintained in DMEM media (11965092, Gibco) containing 10% FBS and 1% P/S. All cell lines were incubated at 37 °C in a humidified incubator with 5% CO_2_. Authentication of the cell lines was regularly performed using short tandem repeat (STR) matching analysis. To establish Reh-6-MP resistant (Reh-6-MPR) and Nalm-6-6-MPR cell lines, the parental Reh and Nalm-6 cells were exposed to 6-MP (HY-13677, MedChemExpress, Shanghai, China) at initial concentrations of 0.5 and 0.25 μg/mL, respectively. After culturing for 48 h, the cells were transferred to a fresh complete medium for an additional 72 h. The process was repeated, and the cells were passaged using gradient-increasing concentrations of 6-MP (0.25 μg/mL –0.5 μg/mL –1 μg/mL –2 μg/mL –4 μg/mL –8 μg/mL –16 μg/mL). The drug-resistant cell lines were successfully established until the cells doubled stably in the medium with 16 μg/mL 6-MP. Before experiments, 6-MP resistant cells would be cultured in the 6-MP-free medium for at least 2 weeks.

### Patient samples

Human bone marrow samples of pediatric patients with B-ALL were collected from Qilu Hospital of Shandong University (Supplementary Tables 1, 2; Jinan, China). Mononucleated cells were isolated using a Ficoll gradient centrifugation method. These primary cells were cultured in RPMI-1640 medium supplemented with 20% FBS and 1% P/S. The protocols in this study were reviewed and approved by the Ethics Committee of Scientific Research of Shandong University Qilu Hospital (KYLL-202306-053) following the tenets of the Declaration of Helsinki. Written informed consent was obtained from all participants.

### Real-time quantitative PCR (RT-qPCR)

Total RNA was extracted using Trizol reagent (15596018, Invitrogen, Carlsbad, CA, USA) or RNA fast assay Kit (220011, Fastagen, Shanghai, China), followed by reverse transcription to cDNA using the ReverTraAce qPCR RT Master Mix kit (FSQ-201, TOYOBO, Osaka, Japan). Quantitative real-time PCR amplification was performed using SYBR Green Realtime PCR Master Mix (QPK-201, TOYOBO) on a Real-Time Thermocycler (qTOWER3G, Analytik Jena AG, Germany), according to the manufacturer’s instructions. Relative fold changes were calculated using the comparative Ct method (2^−ΔΔCt^), with endogenous β-actin serving as a reference gene. All oligonucleotide primers were synthesized by Biosune Biotech (Shanghai, China), and their sequences are listed in Supplementary Table 3.

### Cell viability assays

Cells were seeded in 96-well plates and treated with different doses of 6-MP and histidine (199604, J&K Chemical, Shanghai, China), separately or together. After 48 h of culture, Cell Counting Kit-8 reagent (CCK-8; C6005, New Cell & Molecular Biotech) was added at a volume of 10 μL per well and incubated for an additional 4 h. The optical density value at 450 nm was then measured using a spectrophotometer (Perlong, Beijing, China). Drug sensitivity curves and IC50 were calculated through best-fit analysis of log drug concentration data using GraphPad Prism software version 8.0.

### Colony formation assay

The treated cells were suspended in the complete methylcellulose-based medium (038818 and 04434, Stem Cell Technologies, Vancouver, Canada) and planted into a 12-well plate at a density of approximately 2000–3000 cells/well for Reh-6-MPR and 1 × 10^4^ cells/well for Nalm-6-6-MPR. Following a culture period of 10 to 14 days at 37 °C and 5% CO_2_, colonies consisting of ≥50 cells were manually counted under an inverted microscope (Olympus Tokyo, Japan).

### Cell siRNA and plasmid transfection

The siRNA sequences targeting SIRT5 were as follows: siSIRT5-1, CGUCCACACGAAACCAGAUUU; siSIRT5-2, GAGUCCAAUUUGUCCAGCU. The siRNA was diluted in Opti-MEM (31985070, Gibco) and transfected into cells using Lipofectamine 2000 (11668019, Invitrogen) at a final concentration of 80 nmol/L following the manufacturer’s instructions. The Flag-SIRT5-WT, Flag-SIRT5-H158Y, GFP-HINT1, and respective negative control plasmids were obtained from Miaoling Biology (Wuhan, China) and transfected into 6-MP-resistant leukemia cells with a DNA(μg): lipofectamine 2000 (μL) ratio of 1:2.5. For 293T cells, polyethylenimine (PEI; HY-W250110, MedChemExpress) was employed for plasmids transfection.

### Lentivirus infection

The shSIRT5 lentivirus was packaged by GeneChem Co. (Shanghai, China) using U6-MCS-Ubiquitin-mCherry-puromycin vector and added to Reh-6-MPR and Nalm-6-6-MPR cells at a multiplicity of infection (MOI) of 60 with HiTrans G (GeneChem Co.). Positively infected cells were selected with puromycin (1 μg/mL; ST551, Beyotime, Shanghai, China).

### In vivo experiment

Six to eight-week-old male M-NSG mice (NOD.Cg-Prkdc^scid^Il2rgem1*Smoc*) were obtained from Shanghai Model Organisms Center, Inc. (Shanghai, China). All animal procedures were authorized by the Institutional Animal Care and Use Committee of Shandong University Qilu Hospital (DWLL-2023-069). To establish the murine xenograft model of human B-ALL, stable *SIRT5*-knockdown and control Nalm-6-6-MPR cell lines carrying mCherry fluorescence were transplanted into M-NSG mice intravenously at a dosage of 5 × 10^6^ cells/per mouse. Peripheral blood was collected every 3–4 days to monitor the leukemic progression. Subsequently, 7-day treatment was initiated after detecting more than 1% of human CD19^+^ cells in peripheral blood. For the in vivo experiment depicted in Fig. 2 and Fig. S2, mice were randomly assigned to one of four groups by simple randomization method and treated with PBS (100 μL), histidine (60 mg/kg/day), 6-MP (5 mg/kg/day), or a combination of histidine and 6-MP via tail vein injection. For the animal experiment illustrated in Fig. 6 and Fig. S6, mice were subjected to PBS (100 μL), histidine (60 mg/kg/day) + 6-MP (5 mg/kg/day), or THF (10 mg/kg/day) + histidine + 6-MP through intravenous administration. Leukemia burden was quantified by detecting mCherry fluorescence signal using IVIS spectrum (PerkinElmer, Massachusetts, USA) at the endpoint of treatment. Spleen weight was measured as an indicator of leukemic organ infiltration. Additionally, treatment response was determined by flow cytometric analysis of human CD19^+^ cells among mononucleated cells isolated from the peripheral blood, bone marrow, and spleen samples.

### Flow cytometry analysis

For apoptotic cell determination, samples were washed with cold PBS followed by staining with FITC-Annexin V/propidium iodide (556547, BD Biosciences), or PE-Annexin V/7-Amino-Actinomycin (559763, BD Biosciences) following the manufacturer’s instructions. Cell cycle distribution was examined using propidium iodide (PI; 550825, BD Biosciences) staining. The EdU Cell Proliferation Kit with Alexa Fluor 488 or 647 (CX002 and CX004, CellorLab, Shanghai, China) was utilized to assess the proliferation ability of Reh-6-MPR and Nalm-6-6-MPR cells. The flow cytometry analysis presented in the study was performed with the Guava easyCyte 6HT (Millipore, MA, USA), and the data were evaluated using the Guava Incyte software (Millipore) and Flowjo v10 software.

### LC-MS analysis of intracellular metabolites

The intracellular metabolite was extracted from 5 × 10^6^ treated cells using ice-cold 80% methanol containing 0.5% ascorbic acid (1043003, Merck, Darmstadt, Germany) and 0.5% 2-mercaptoethanol (BME; 21985-023, Gibco). After thorough mixing by vortexing on ice and centrifugation at 16,000×*g* for 15 min at 4 °C, the supernatant was transferred to a new tube and dried using the Integrated SpeedVac System (Thermo Fisher, Waltham, MA, USA). For metabolites profiling, the dried samples were resuspended in 200 μL of a solution containing 0.1% formic acid (5330020050, Merck) and loaded onto a C18 HPLC column (2.2 μm particle size, 2.1 × 100 mm, Thermo Fisher), which was coupled online to Dionex UltiMate 3000 UPLC system (Thermo Fisher) operating in full-scanned positive electrospray ionization mode. The mobile phase was composed of 0.1% formic acid (buffer A) and acetonitrile (51101, Thermo Fisher) containing 0.1% formic acid (buffer B). The flow rate was set at 0.4 mL/min with the following gradient: 0–5 min, held at 5% B; 5–10 min, a linear increase from 5 to 20% of B; 10.1–14.0 min, linear gradient from 36 to 95% B; 14.1–18.0 min, the gradient was brought back to initial conditions and maintained [19]. Relative quantitation of intracellular metabolites was performed using Compass DataAnalysis 4.4 (Bruker, Bremen, Germany) with a 20ppm mass tolerance.

### RNA-sequencing and analysis

Reh-6-MPR cells were treated with PBS, histidine (4 mM), 6-MP (1.0 μg/mL), or a combination of histidine and 6-MP for 48 h, and then immediately collected with Trizol reagent (Invitrogen) for total RNA extraction. The collected samples were then commissioned to sequence, quantify, and analyzed by Xiuyue Biol (Jinan, China). Differentially expressed genes (DEGs) were identified using DESeq2, with criteria of absolute log2 fold change ≥1 and adjusted *P* value <0.05. The original data are available in the GEO repository under the accession number GSE236934.

### Western blot

The cell lysates were separated via sodium dodecyl sulfate-polyacrylamide gel electrophoresis (SDS-PAGE) and transferred onto polyvinylidene fluoride (PVDF) membranes (ISEQ00010, Millipore). Subsequently, the membranes were blocked with 5% skimmed milk and incubated overnight at 4 °C with primary antibodies. The following antibodies were used: anti-SIRT5 (1:1000 dilution; 8782 S, Cell Signaling Technology, Danvers, MA, USA), anti-HINT1 (1:1000 dilution; 67583-1-Ig, Proteintech, Rosemont, IL, USA), anti-Succinyllysine (1:1000 dilution; PTM-401, PTM Bio, Hangzhou, China), anti-Acetyllysine (1:500 dilution; PTM-105RM, PTM Bio), anti-GFP (1:5000 dilution; 50430-2-AP, Proteintech), anti-Flag (1:5000 dilution; 80010-1-RR, Proteintech), and anti-β-Actin (1:5000 dilution; AB0035, Abways, Shanghai, China). Then PVDF membranes were incubated with HRP-conjugated goat anti-rabbit or mouse antibodies (1:5000 dilution; AB0101 and AB0102, Abways) and thoroughly washed. The protein bands were visualized using the ECL chemiluminescence detection kit (WBKLS0500, Millipore), and band intensity was quantified using ImageJ software.

### Immunoprecipitation (IP) and Co-IP

Cells were lysed with 1% NP-40 buffer (P0013F, Beyotime) with freshly added proteinase and phosphatase inhibitor cocktail. Then the lysate was incubated with Flag Fab-Trap™ Agarose (ffa, Chromotek, Proteintech) or Anti-GFP Beads (L-1016, Biolinkedin, Shanghai, China) with gentle rotation overnight at 4 °C. The captured complexes were washed, boiled at 95 °C in SDS sample buffer, and detected by SDS-PAGE. To investigate the SIRT5-interacting proteins, the gels were stained with coomassie brilliant blue (CBB) and cut for mass spectrometry analysis, which was performed by PTM Bio. The interaction between HINT1 and SIRT5, as well as the acetylation or succinylation of HINT1, were further validated by Western blot using corresponding antibodies.

### Statistical analysis

Before conducting comparisons, the quantitative data were examined for normality using the Kolmogorov–Smirnov test, followed by the variance homogeneity test using the Bonferroni test. Statistical analysis was performed using GraphPad Prism 8.0.1 and results are represented as mean ± SEM. An unpaired two-tailed *t*-test was conducted to analyze the differences between the two groups. One-way analysis of variance (ANOVA) with Dunnett’s test was conducted to compare among multiple homogeneous groups. Comparisons of grouped data were calculated using two-way ANOVA with Tukey’s test. Nonparametric Spearman’s correlation analysis was performed to create correlation matrices. Results with *P* values less than 0.05 were considered statistically significant. The selection of sample size was based on extensive previous studies. No data was excluded from the study.

### Supplementary information

Supplementary Figures and legends

Supplementary Tables

Original western blots

Reproducibility checklist

## Data Availability

The RNA-seq data has been deposited in the Gene Expression Omnibus (GEO) repository under the accession number GSE236934. The original western blot data has also been placed in the supplementary material. Any additional information required to reanalyze the data reported in this paper is available from the lead contact upon reasonable request.
