# Supplementary material for: Histidine re-sensitizes pediatric acute lymphoblastic leukemia to 6-mercaptopurine through tetrahydrofolate consumption and SIRT5-mediated desuccinylation
Source: Cell Death Dis. 2024 Mar 14;15(3):216. doi: 10.1038/s41419-024-06599-5 (PMC10940622; doi:10.1038/s41419-024-06599-5)
Supplement: Supplementary file 1 — Supplementary Figures and legends [file 41419_2024_6599_MOESM1_ESM.docx]

**Histidine Re-sensitizes Pediatric Acute Lymphoblastic Leukemia to 6-Mercaptopurine Through Tetrahydrofolate Consumption and SIRT5-mediated Desuccinylation**

**Supplementary Figures**


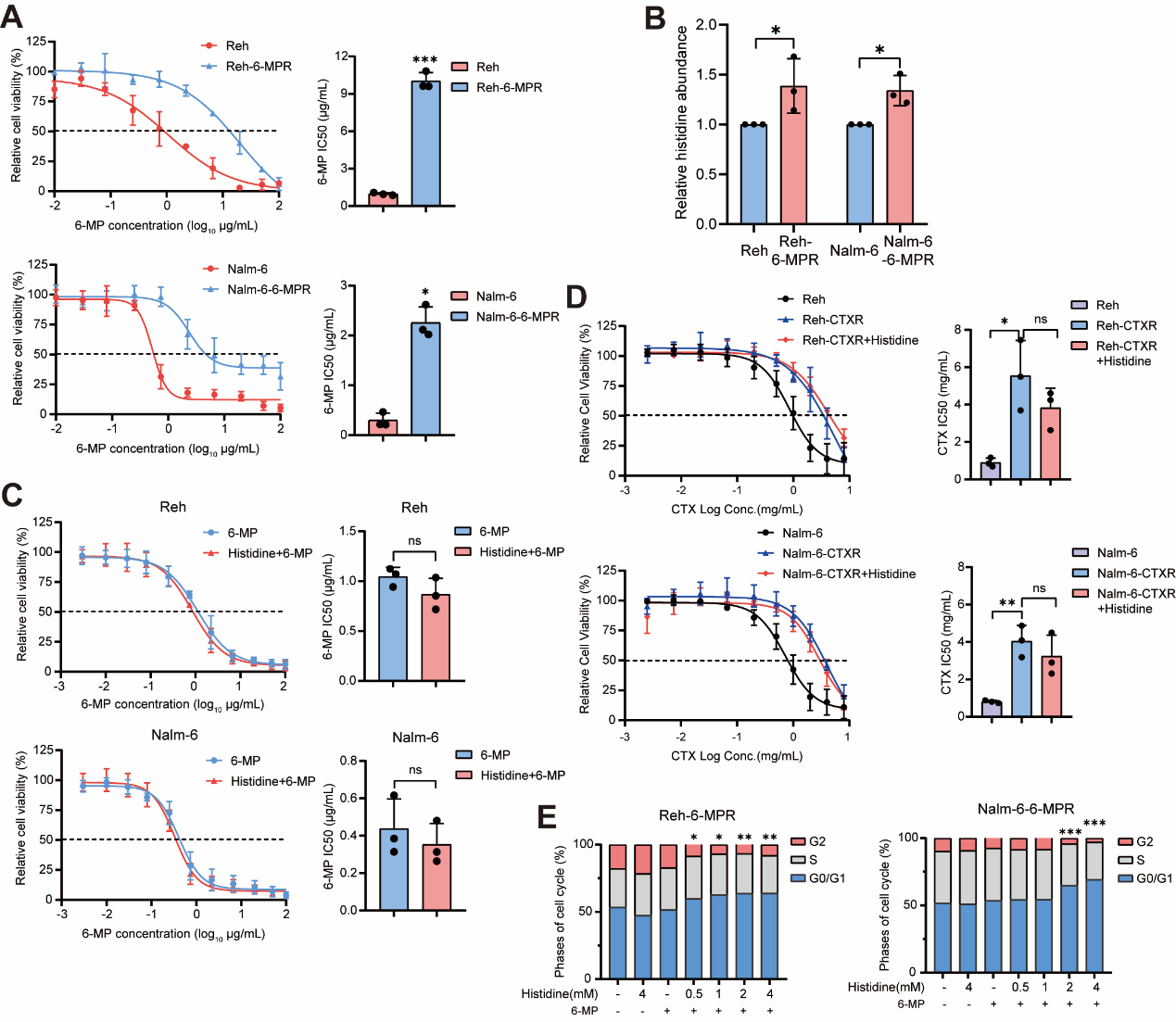


## Supplementary Fig. 1 Role of histidine in the chemosensitivity of B-ALL cells.

**A** Drug sensitivity curves and IC50 values of Reh-6-MPR, Nalm-6-6-MPR cells, and their respective parental cell line (n=3). **B** Intracellular histidine abundance in Reh-6-MPR cells, Nalm-6-6-MPR cells, and their corresponding parental cell lines measured by LS-MS (n=3). **C**–**D** Drug sensitivity curves and IC50 values after treatment with increasing concentrations of 6-MP (**C**) or CTX (**D**), in the presence or absence of histidine (4mM) for 48 hours (n=3). **E** Flow cytometric analysis of cell cycle of Reh-6-MPR and Nalm-6-6-MPR cells treated with the indicated agents for 48 h. 6-MP was applied at a concentration of 1.0μg/mL in Reh-6-MPR and 0.5μg/mL in Nalm-6-6-MPR cells (n=3). Data are presented as mean ± SEM. **P* < 0.05, ***P* < 0.01, ****P* < 0.005.


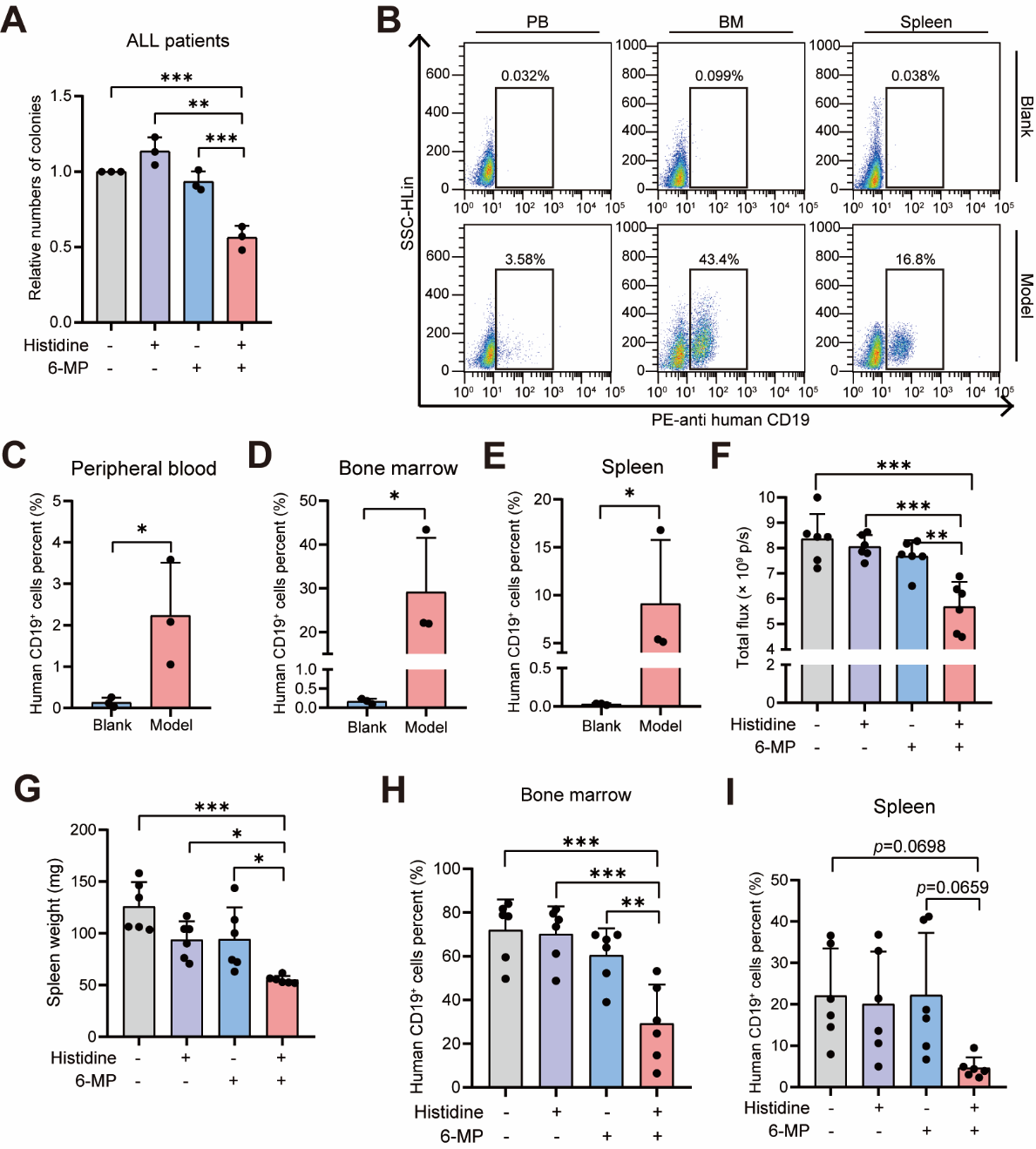


## Supplementary Fig. 2 Establishment of human B-ALL xenotransplantation mouse model.

**A** Quantification of the colony formation assay in primary B-ALL cells treated with PBS, histidine, 6-MP, or a combination of histidine and 6-MP (n=3). **B**–**E** Representative flow cytometric images (**B**) and quantification of the percentage of human CD19^+^ B-ALL cells in peripheral blood (**C**), bone marrow (**D**), and spleen (**E**) at D14 in mice with or without Nalm-6-6-MPR transplantation (n=3). **F** Quantification of tumor burden monitored by detecting up the mCherry signal via IVIS spectrum of mice engrafted with Nalm-6-6-MPR cells at the endpoint of treatment (n=6). **G** Weights of spleens isolated from mice at the endpoint of indicated treatment (n=6). **H**–**I** Quantification of human CD19^+^ cells in the bone marrow (**H**) and spleen (**I**) from xenotransplanted mice at the endpoint of treatment (D21, n=6). Data are presented as mean ± SEM. **P* < 0.05, ***P* < 0.01, ****P* < 0.005.


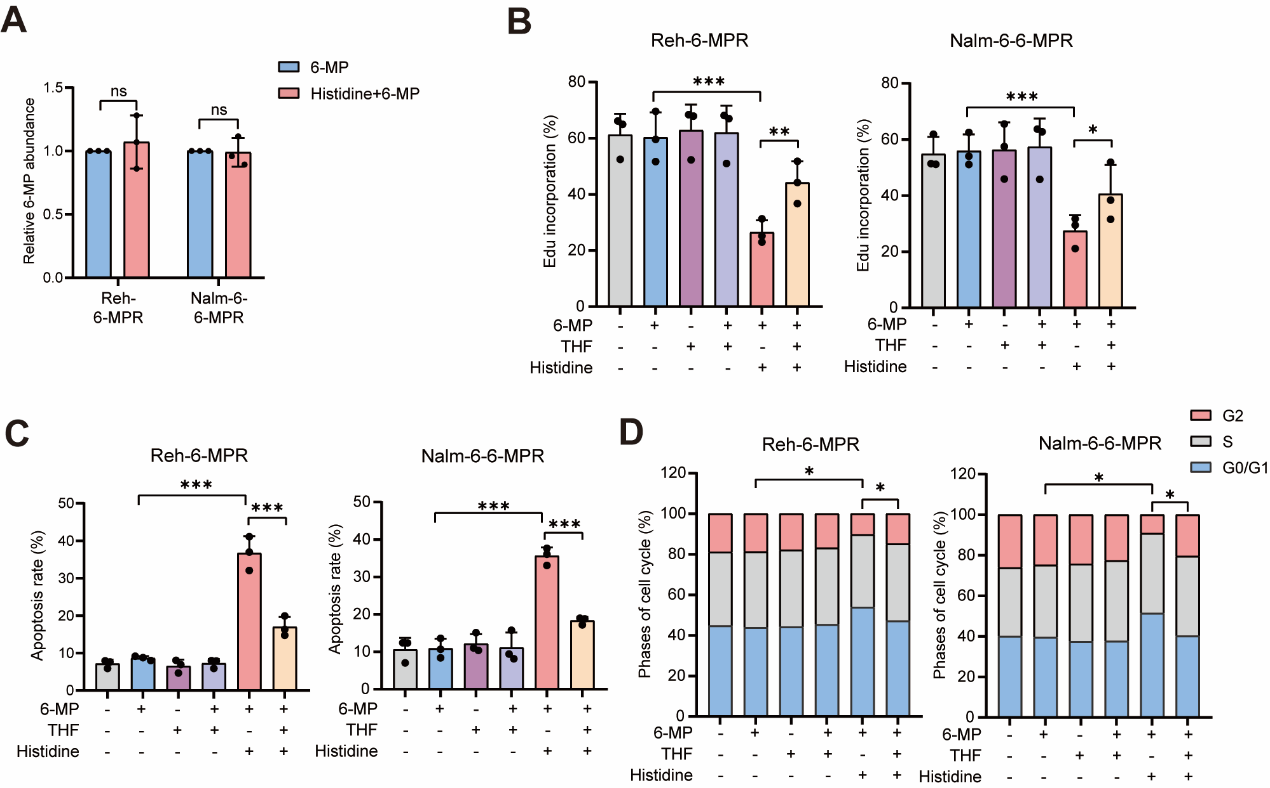


## Supplementary Fig. 3 The role of THF intervention in histidine-treated leukemia cells in the presence of 6-MP.

**A** Intracellular 6-MP levels in Reh-6-MPR and Nalm-6-6-MPR cells treatment with 6-MP alone or in combination with histidine measured via LS-MS (n=3). **B** Quantification of cell proliferation ratio of Reh-6-MPR and Nalm-6-6-MPR cells, measured by EdU assay with Alexa Fluor 488 staining (n=3). **C** Statistical results of apoptotic analysis Reh-6-MPR and Nalm-6-6-MPR cells treated with the indicated agents for 48 h, measured by FITC-Annexin V/PI staining (n=3). **D** Flow cytometric analysis of cell cycle and the percentages of G0/G1, S, and G2/M-phase in Reh-6-MPR and Nalm-6-6-MPR cells treated with the indicated agents for 48 h (n=3). Data are presented as mean ± SEM. **P* < 0.05, ***P* < 0.01, ****P* < 0.005.


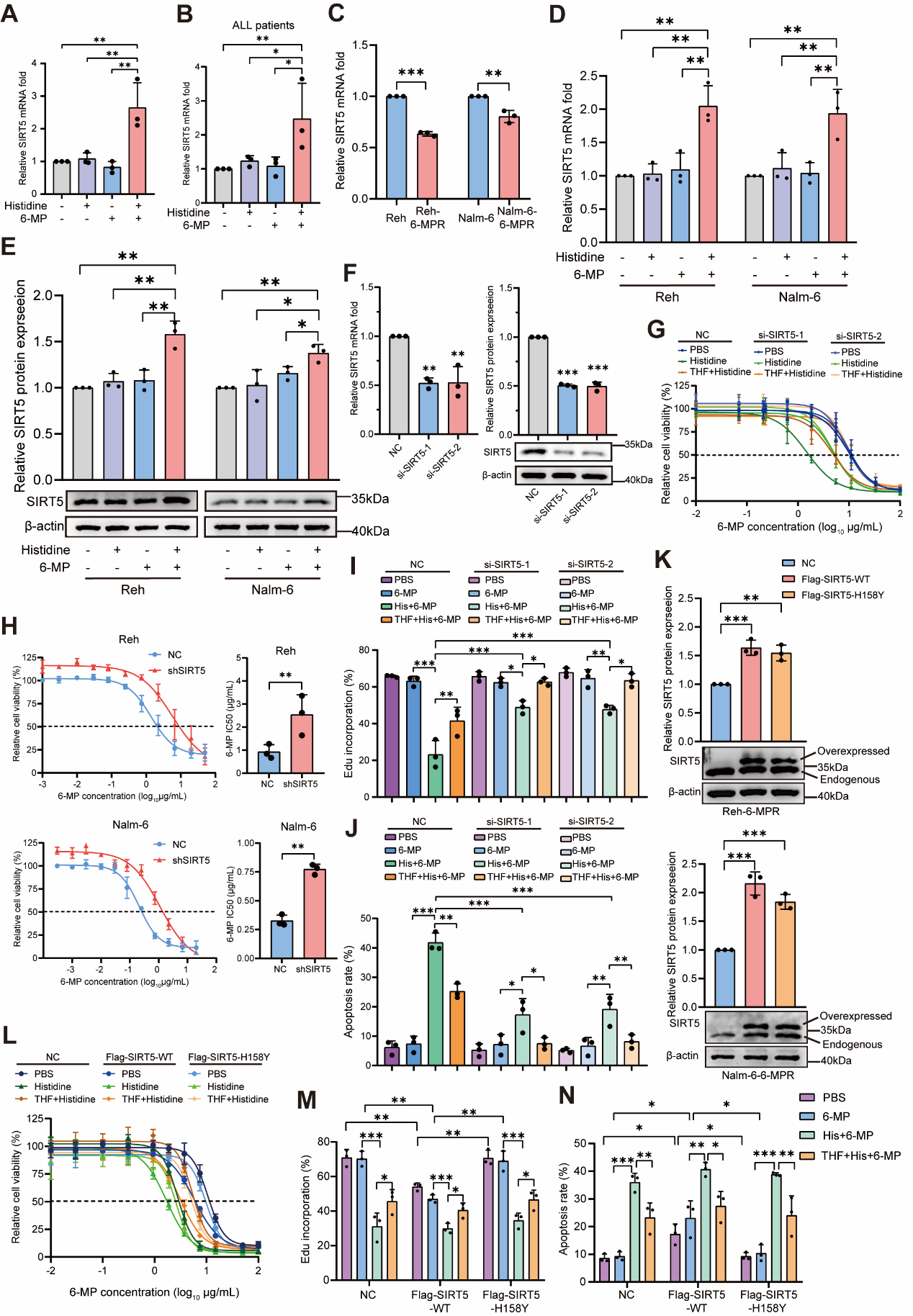


## Supplementary Fig. 4 SIRT5 cooperated with THF consumption to account for the inhibitory effects of the combination of 6-MP and histidine on leukemia cells.

**A**–**B** Validation of upregulated *SIRT5* mRNA expression in Nalm-6-6-MPR cells (**A**; n=3) and primary B-ALL samples (**B**; n=3) after being treated with both histidine and 6-MP. **C** Comparison of SIRT5 mRNA expression between 6-MP resistant leukemia cell lines and their relevant parental strains (n=3). **D**–**E** SIRT5 mRNA (**D**) and protein (**E**) expression in Reh and Nalm-6 cells treated with histidine (4mM), 6-MP (0.25μg/mL in Reh and 0.1μg/mL in Nalm cells), or a combination of histidine and 6-MP for 48h (n=3)**. F** Inhibition of SIRT5 mRNA (left) and protein (right) expression by two siRNAs targeting SIRT5, as verified by RT-qPCR and western blot (n=3). **G** Drug sensitivity curves of 6-MP in SIRT5-downregulated and control 6-MP resistant leukemia cells after treatment with increasing concentrations of 6-MP (0–100µg/mL), in the presence of PBS, histidine (4mM), or a combination of THF (7.5μM) and histidine for 48 hours (n=3). **H** Drug sensitivity curves and IC50 values of Reh cells, Nalm-6 cells, and their respective cell lines with SIRT5 knocked-down (n=3). **I**–**J** Quantitative analysis of cell proliferation ratio (**I**) and apoptotic proportion (**J**) in SIRT5-downregulated and control leukemia cells treated with the indicated agents for 48 h (n=3). **K** Western blot verification of the establishment of cell lines overexpressing SIRT5 wild type (SIRT5-WT) and SIRT5-H158Y. **L** Drug sensitivity curves of 6-MP in SIRT5-WT/ SIRT5-H158Y-overexpressed and control leukemia cells (n=3). **M**–**N** Quantitative analysis of cell proliferation ratio (**M**) and apoptotic proportion (**N**) in SIRT5-WT/ SIRT5-H158Y-overexpressed and control leukemia cells treated with the indicated agents for 48 h (n=3). Data are presented as mean ± SEM. **P* < 0.05, ***P* < 0.01, ****P* < 0.005.


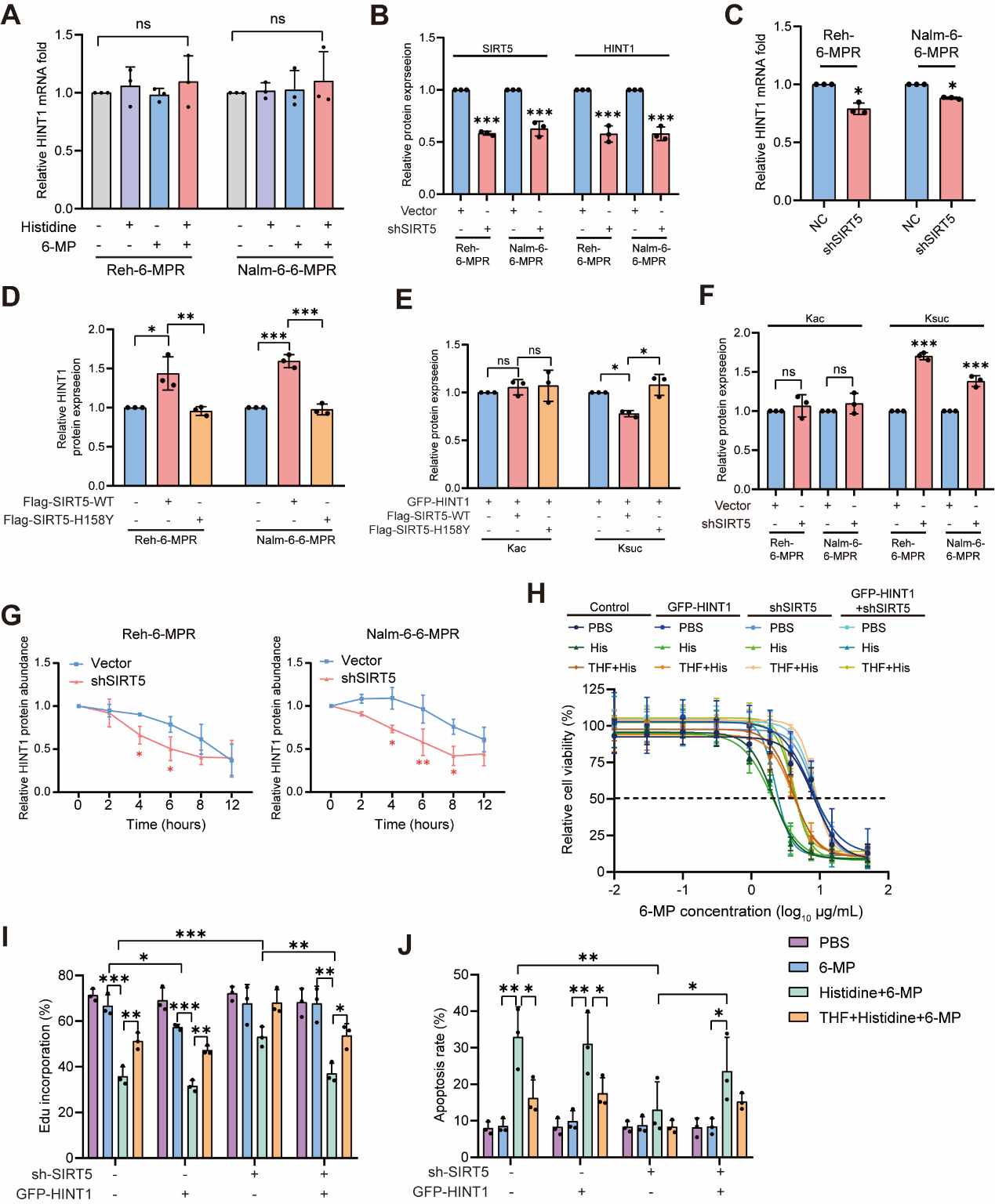


## Supplementary Fig. 5 SIRT5 de-succinylated HINT1 to exert an anti-leukemic effect.

**A** Quantification of HINT1 mRNA levels in Reh-6-MPR and Nalm-6-6MPR cells treated with histidine and 6-MP (n=3). **B**–**C** Quantification of HINT1 protein (**B**) and mRNA levels (**C**) in Reh-6-MPR and Nalm-6-6MPR cells with SIRT5 knocked-down using lentivirus (n=3). **D** Quantification of HINT1 protein in Reh-6-MPR and Nalm-6-6MPR cells with SIRT5 overexpressed by plasmid transfection (n=3). **E**–**F** Quantification of the acetylation and succinylation levels of GFP-bead-immunoprecipitated HINT1 in SIRT5-overexpressed (SIRT5-WT or SIRT5-H158Y) 293T cells (**E**; n=3) and SIRT5-knocked-down leukemia cells (**F**; n=3). **G** Assessment of HINT1 half-lives in Reh-6-MPR and Nalm-6-6MPR cells (n=3). **H** Drug sensitivity curves of 6-MP in HINT-overexpressed and/or SIRT5-knocked-down leukemia cells (n=3). **I**–**J** Quantitative analysis of cell proliferation ratio (**I**) and apoptotic proportion (**J**) in HINT-overexpressed and/or SIRT5-knocked-down leukemia cells treated with the indicated agents for 48 h (n=3). Data are presented as mean ± SEM. **P* < 0.05, ***P* < 0.01, ****P* < 0.005. Ksuc, succinyllysine; Kac, acetyllysine.


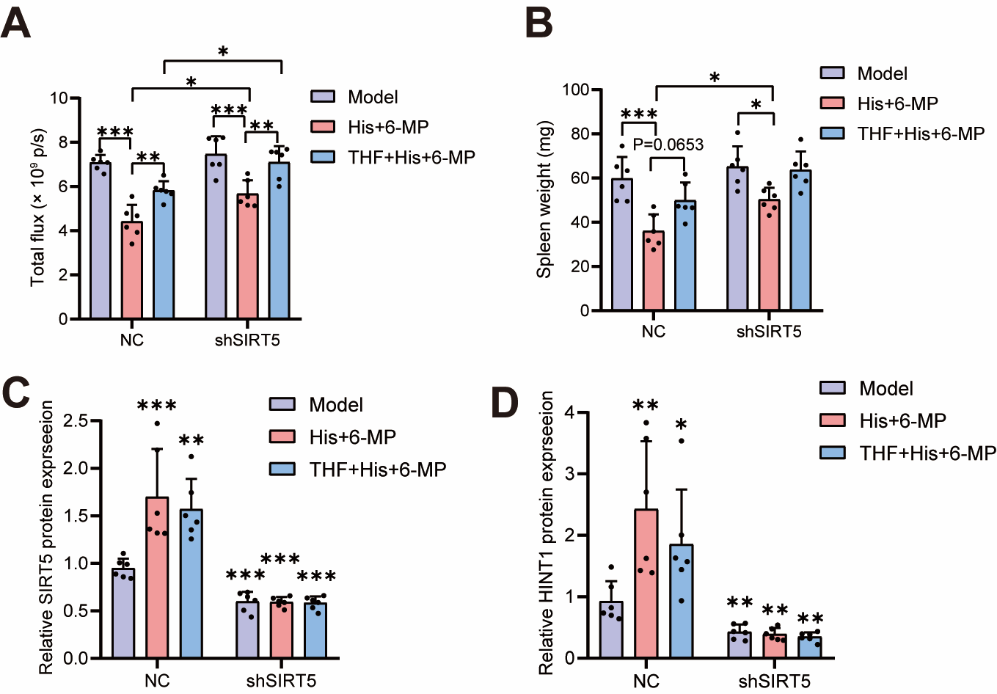


## Supplementary Fig. 6 Knockdown of SIRT5 and THF supplementation significantly impairs the chemo-sensitization efficacy of histidine *in vivo*. A Quantification of leukemia burden monitored by detecting the mCherry signal via IVIS spectrum in mice engrafted with SIRT5-knockdown or control Nalm-6-6MPR cells at the endpoint of treatment (n=6). B Weight of spleens isolated from mice bearing SIRT5-knockdown or control cells at the endpoint of treatment (n=6). C–D Quantification of SIRT5 (C) and HINT1 (D) protein expression in bone marrow derived from mice bearing SIRT5-knockdown or control Nalm-6-6-MPR cells (n=6). Data are presented as mean ± SEM. **P* < 0.05, ***P* < 0.01, ****P* < 0.005.
