# Supplementary material for: Histidine re-sensitizes pediatric acute lymphoblastic leukemia to 6-mercaptopurine through tetrahydrofolate consumption and SIRT5-mediated desuccinylation
Source: Cell Death Dis. 2024 Mar 14;15(3):216. doi: 10.1038/s41419-024-06599-5 (PMC10940622; doi:10.1038/s41419-024-06599-5)
Supplement: Supplementary file 2 — Supplementary Tables [file 41419_2024_6599_MOESM2_ESM.docx]

**Histidine Re-sensitizes Pediatric Acute Lymphoblastic Leukemia to 6-Mercaptopurine Through Tetrahydrofolate Consumption and SIRT5-mediated Desuccinylation**

## Supplementary Table 1. The basic characteristics and relative histidine abundance of children newly diagnosed with B-ALL.

| **Patient number** | **Gender** | **Age**  **(months)** | **MRD46**  **(%)** | **Sample state** | **Normalized histidine abundance** |
| --- | --- | --- | --- | --- | --- |
| 1 | Male | 54 | <0.03 | Refractory | 1.328489588 |
| 2 | Male | 75 | <0.01 | Remission | 1.255910449 |
| 3 | Male | 31 | <0.01 | Remission | 1.162109053 |
| 4 | Male | 34 | <2.05 | Refractory | 1.004413188 |
| 5 | Female | 95 | <0.01 | Remission | 0.869955176 |
| 6 | Male | 117 | <0.58 | Refractory | 0.846317122 |
| 7 | Male | 85 | <0.01 | Remission | 0.826762191 |
| 8 | Male | 45 | <0.01 | Remission | 0.790001839 |
| 9 | Female | 58 | <0.14 | Refractory | 0.785795278 |
| 10 | Male | 48 | <0.01 | Remission | 0.713813347 |
| 11 | Female | 51 | <0.01 | Remission | 0.692389835 |
| 12 | Male | 137 | <0.01 | Remission | 0.652877406 |
| 13 | Female | 82 | <0.01 | Remission | 0.601194518 |
| 14 | Male | 25 | <0.63 | Refractory | 0.585696403 |
| 15 | Female | 97 | <0.01 | Remission | 0.542669883 |
| 16 | Male | 39 | <0.01 | Remission | 0.346973305 |
| 17 | Male | 15 | <0.01 | Remission | 0.309997308 |
| 18 | Female | 169 | <0.01 | Remission | 0.289751337 |
| 19 | Male | 29 | <0.01 | Remission | 0.198252938 |
| 20 | Male | 100 | <0.01 | Remission | 0.183203039 |
| 21 | Female | 28 | <0.01 | Remission | -0.115452142 |
| 22 | Male | 62 | <0.01 | Remission | -0.19920625 |
| 23 | Male | 42 | <0.01 | Remission | -0.408580048 |
| 24 | Female | 41 | <0.01 | Remission | -0.520093054 |
| 25 | Female | 29 | <0.01 | Remission | -0.666304446 |

## Supplementary Table 2. The characteristics of primary B-ALL samples.

| **Patient number** ^a)^ | **Gender** | **Age** | **Sample state** | **WBC count (×10^9^/mL)** |
| --- | --- | --- | --- | --- |
| 1 | Female | 1y11m | Remission | 37.29 |
| 2 | Male | 12y8m | Remission | 5.5 |
| 3 | Male | 11y6m | Remission | 215.52 |
| 4 | Male | 1y6m | Remission | 296.95 |
| 5 | Male | 10y4m | Remission | 122.58 |
| 6 | Female | 8y9m | Remission | 40.2 |
| 7 | Male | 7y7m | Remission | 11.81 |
| 8 | Male | 4y7m | Remission | 11.07 |
| 9 | Male | 13y9m | Remission | 7.55 |
| 10 | Female | 2y8m | Remission | 76.44 |
| 11 | Female | 13y3m | Remission | 52.43 |
| 12 | Female | 3y11m | Remission | 6.5 |
| 13 | Male | 11y6m | Refractory | 215.52 |
| 14 | Male | 2y10m | Refractory | 32.57 |
| 15 | Male | 10y1m | Refractory | 78.79 |
| 16 | Male | 7y8m | Refractory | 4.2 |
| 17 | Female | 8y7m | Refractory | 27.63 |
| 18 | Female | 4y7m | Refractory | 2.11 |
| 19 | Male | 3y4m | Refractory | 241.98 |
| 20 | Female | 3y3m | Refractory | 14.35 |
| 21 | Male | 9y10m | Refractory | 13.16 |
| 22 | Male | 11y2m | Refractory | 2.68 |
| 23 | Female | 10y2m | Refractory | 97.51 |
| 24 | Female | 4y4m | Refractory | 20.78 |
| 25 | Female | 13y6m | Relapse | 175.09 |
| 26 | Male | 3y | Relapse | 98.86 |
| 27 | Male | 9y | Relapse | 71.1 |
| 28 | Male | 4y3m | Relapse | 8.66 |
| 29 | Male | 5y5m | Relapse | 80.93 |
| 30 | Male | 8y | Relapse | 274.31 |
| 31 | Female | 4y7m | Relapse | 6.58 |
| 32 | Female | 5y5m | Relapse | 7.18 |
| 33 | Male | 14y | Relapse | 22.05 |
| 34 | Female | 11y10m | Relapse | 302.25 |
| 35 | Female | 2y9m | Relapse | 77.9 |
| 36 | Female | 5y6m | Relapse | 0.85 |
| 37 | Male | 13y7m | Relapse | 42.94 |
| 38 | Male | 2y10m | Relapse | 9.45 |
| 39 | Male | 8y | Relapse | 55.74 |

Abbreviations: y, year(s); m, month(s); WBC; white blood cell.

^a)^ No. 1-36 are used for RT-qPCR analysis in Fig. 1C; No. 26, 30, and 35 are used for 6-MP sensitivity analysis (Fig. 2A); No. 37-39 are used for colony formation assay in Fig. 2B and expression examination in Fig. 3B, 4E. No. 4-9, 32-34, and 37-39 are used for western-blot analysis in Fig. 4G.

## Supplementary Table 3. Primer sequences for RT-qPCR.

| **Genes** | | **Sequence (5′– 3′)** | **Length (bp)** |
| --- | --- | --- | --- |
| HAL | Forward primer | TGGCTGGGCTGATGCTAAAT | 20 |
|  | Reverse primer | CAATAGCACTGGCTCGCTCT | 20 |
| UROC | Forward primer | GAGGCAAGTCTCAGCCATCA | 20 |
|  | Reverse primer | GCACATAGGAAGGGTAGCGG | 20 |
| AMDHD | Forward primer | GTGGATATGGCCTCGACCTG | 20 |
|  | Reverse primer | CGCAGTAGGTAGCCGAGATG | 20 |
| FTCD | Forward primer | GTGGGCCTCATGACCTACGG | 20 |
|  | Reverse primer | CCTCATTGCTTCCAGGTAGGC | 21 |
| SIRT5 | Forward primer | CGACCTCTCCAGATTGTCCCA | 21 |
|  | Reverse primer | CTGGTTTCGTGTGGACGCTG | 20 |
| β-actin | Forward primer | GAAGAGCTACGAGCTGCCTGA | 21 |
|  | Reverse primer | CAGCCAGCACTGTGTTGGCG | 20 |
