# Supplementary figures and images for: Histidine re-sensitizes pediatric acute lymphoblastic leukemia to 6-mercaptopurine through tetrahydrofolate consumption and SIRT5-mediated desuccinylation
Source: Cell Death Dis. 2024 Mar 14;15(3):216. doi: 10.1038/s41419-024-06599-5 (PMC10940622; doi:10.1038/s41419-024-06599-5)

**Original images of representative Western-blot**


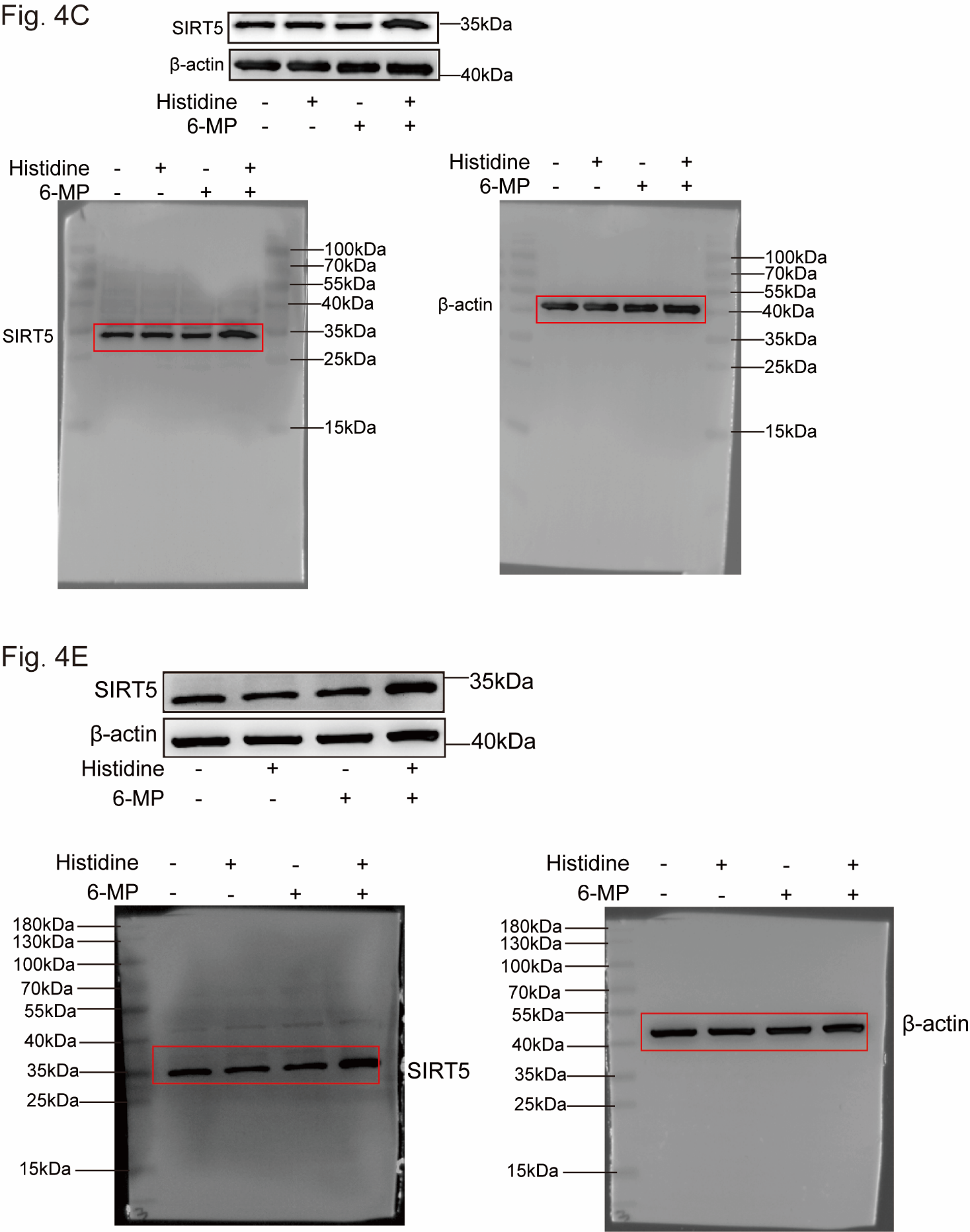


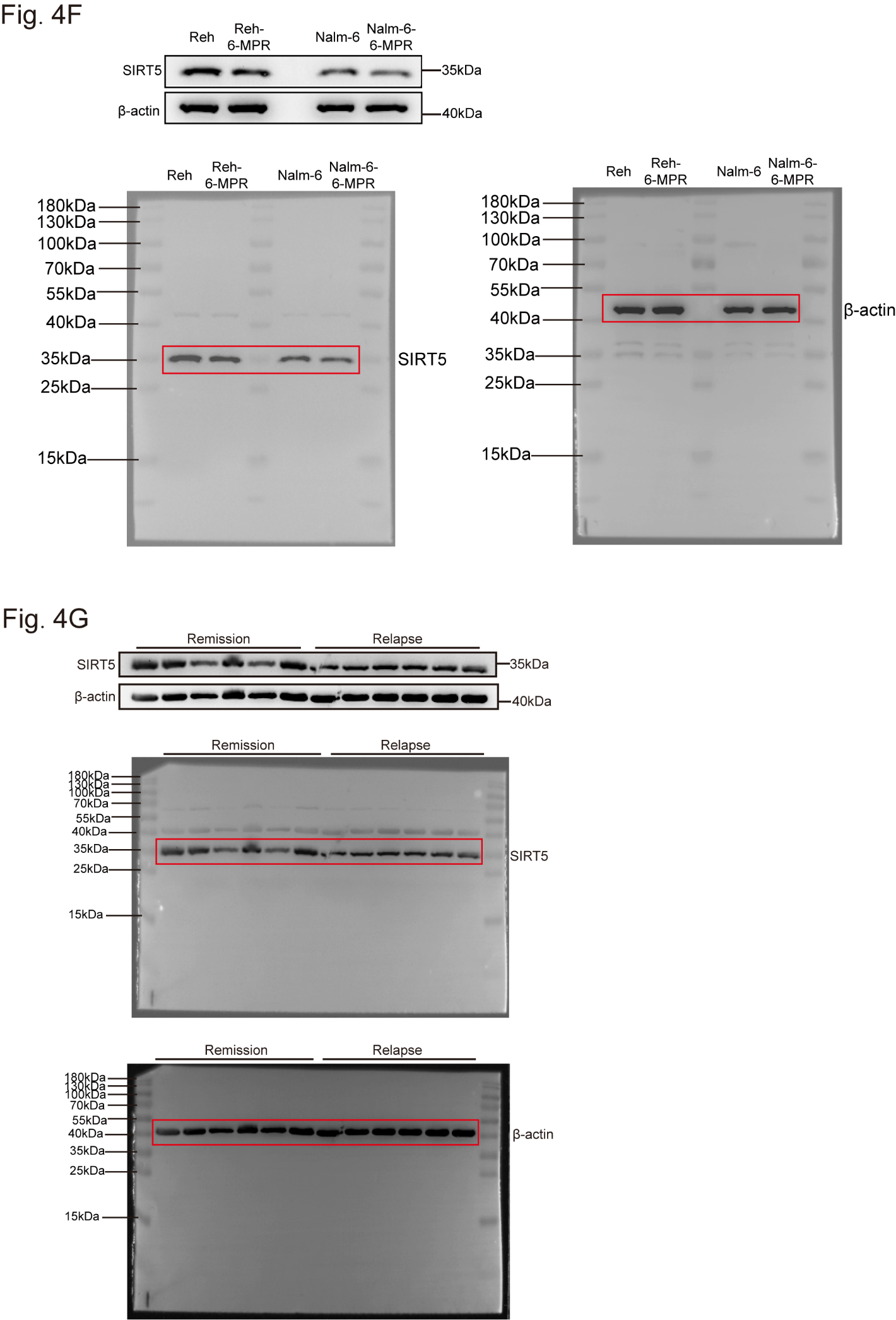


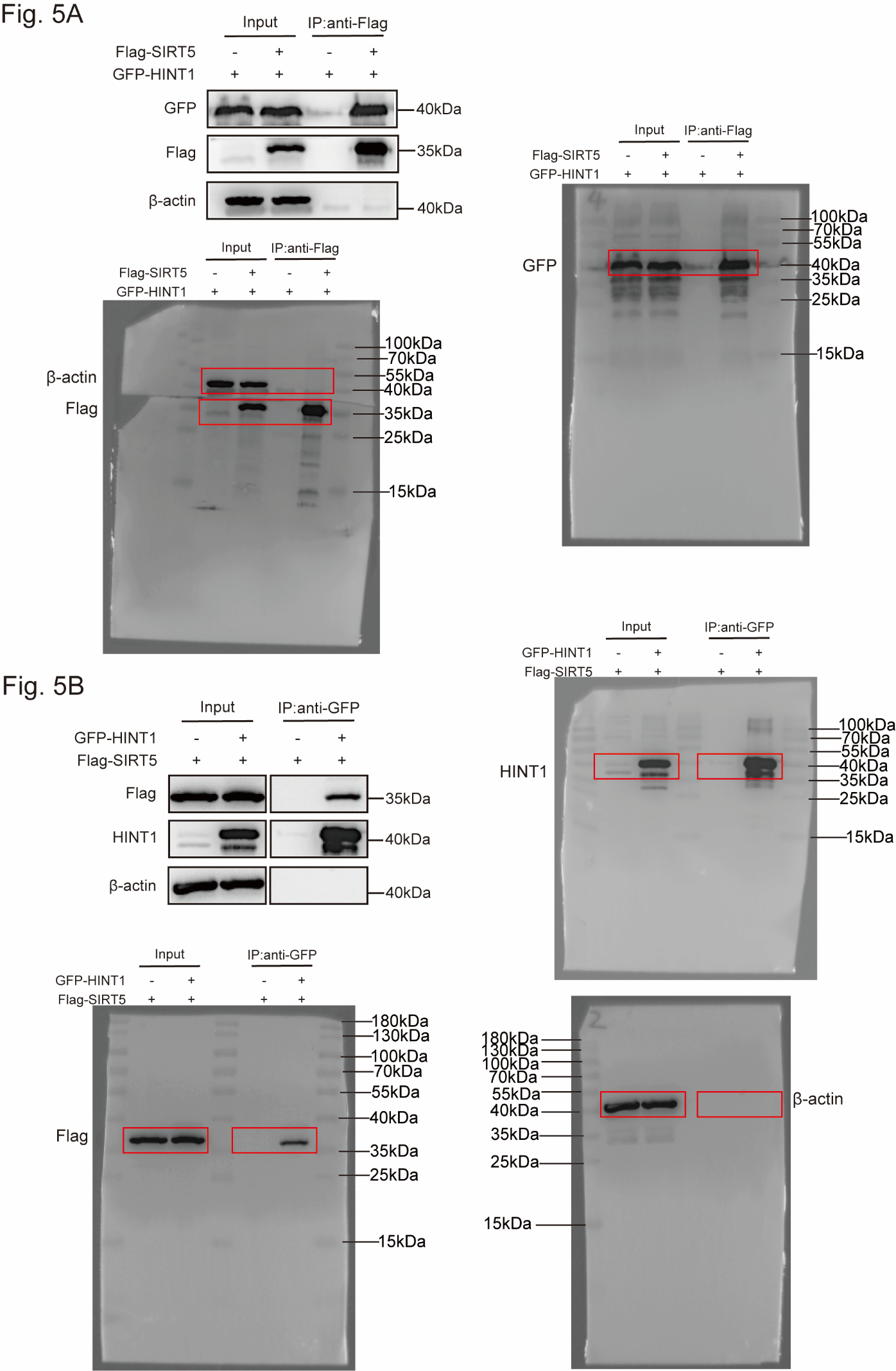


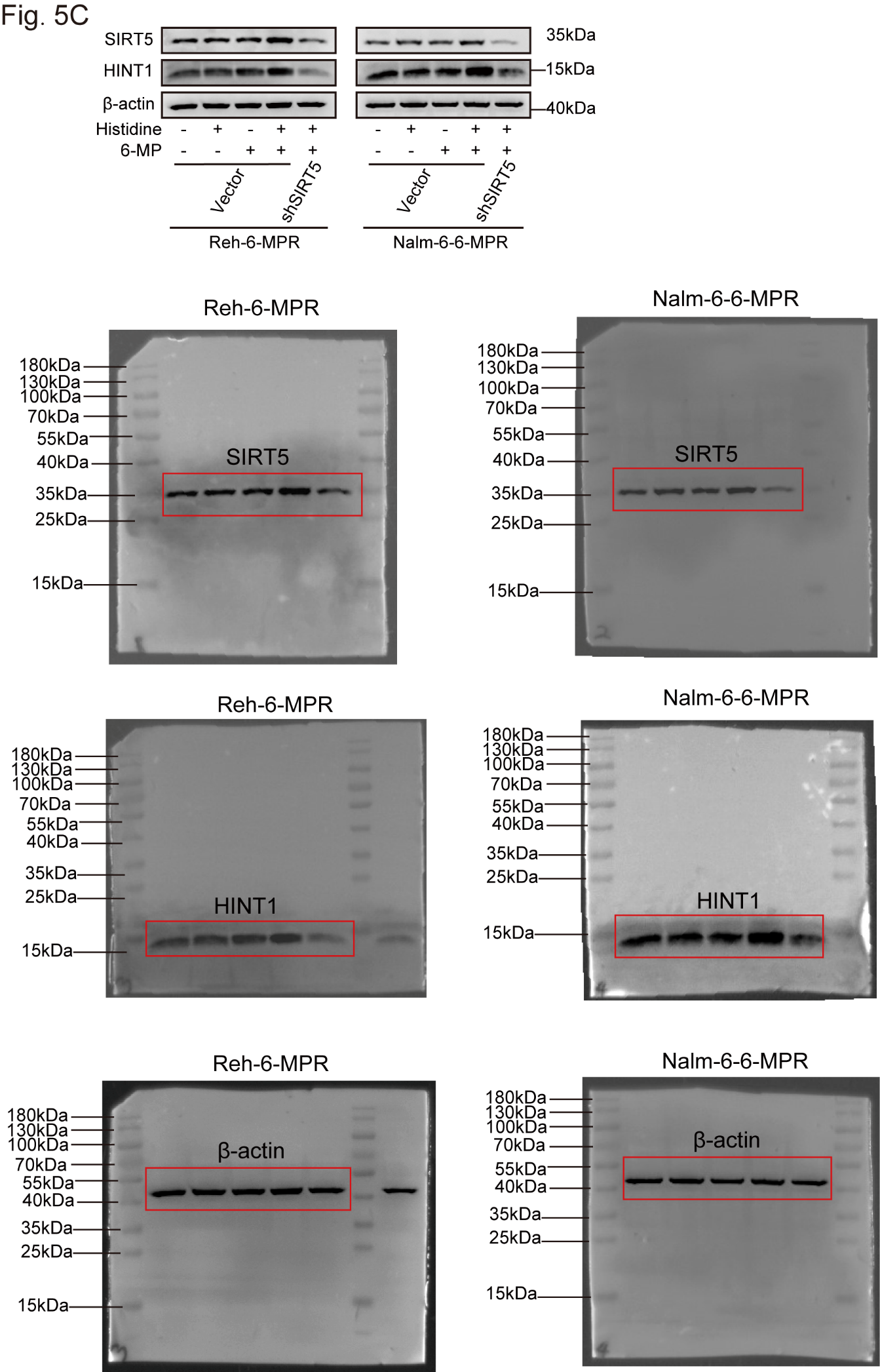


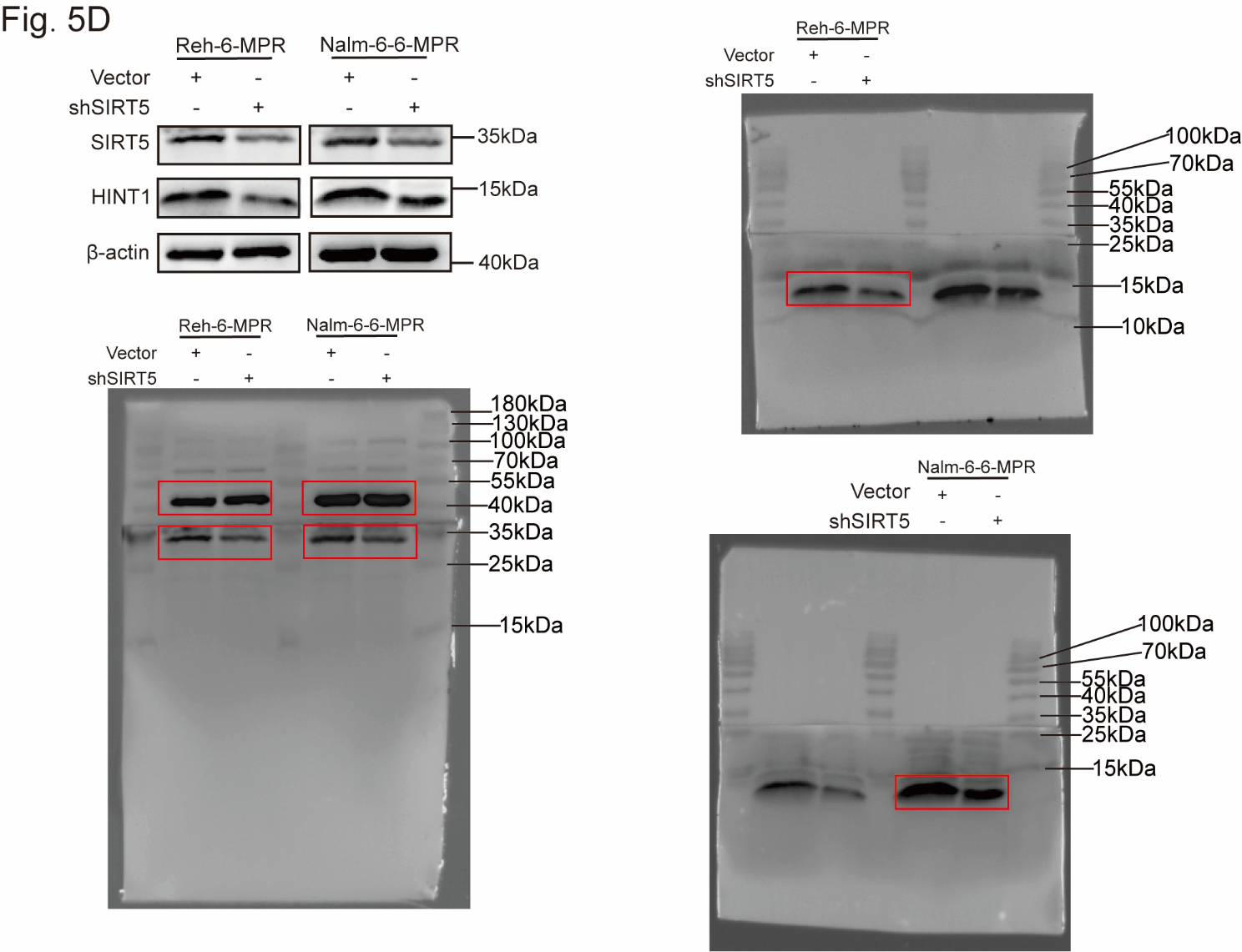


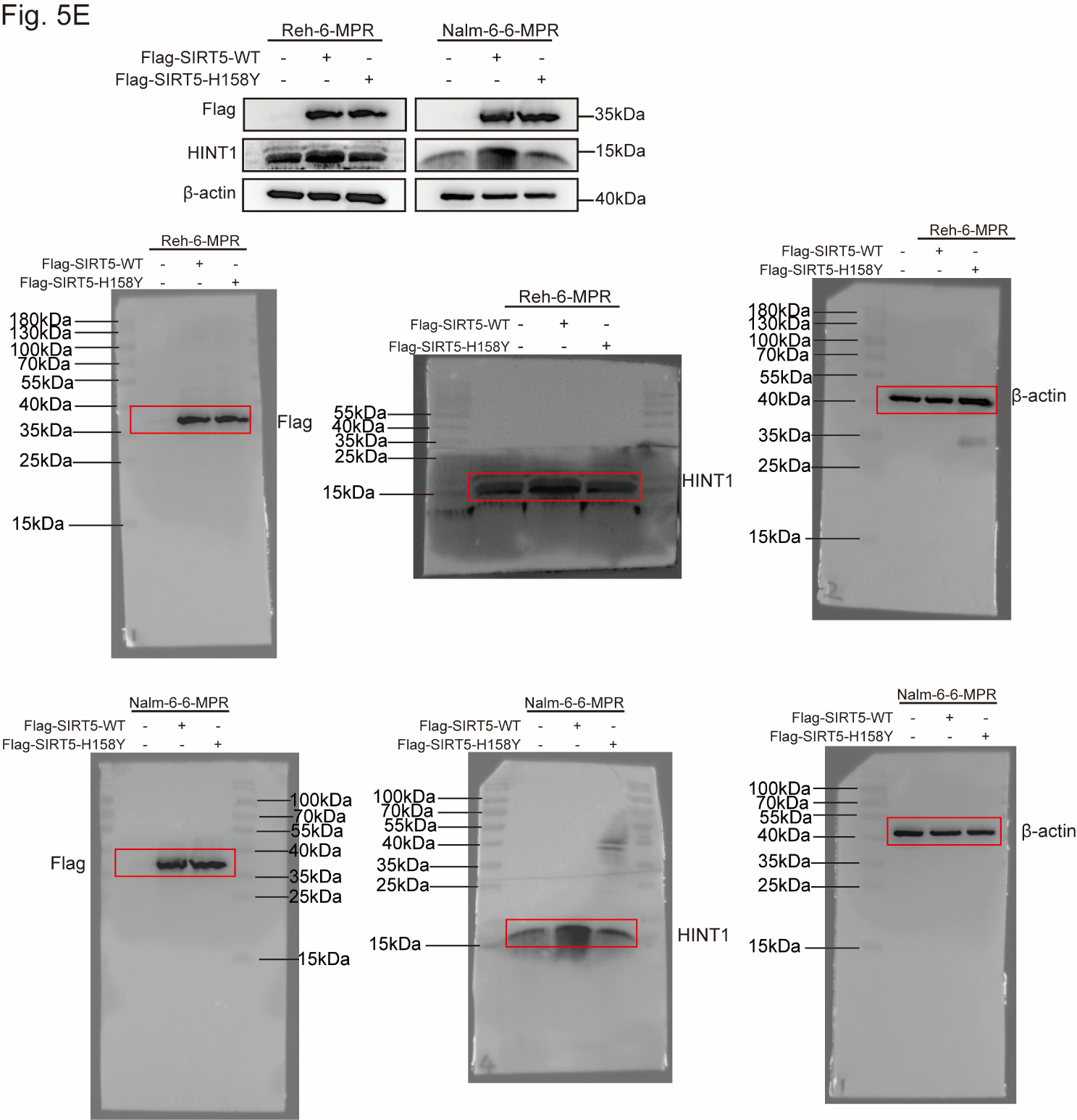


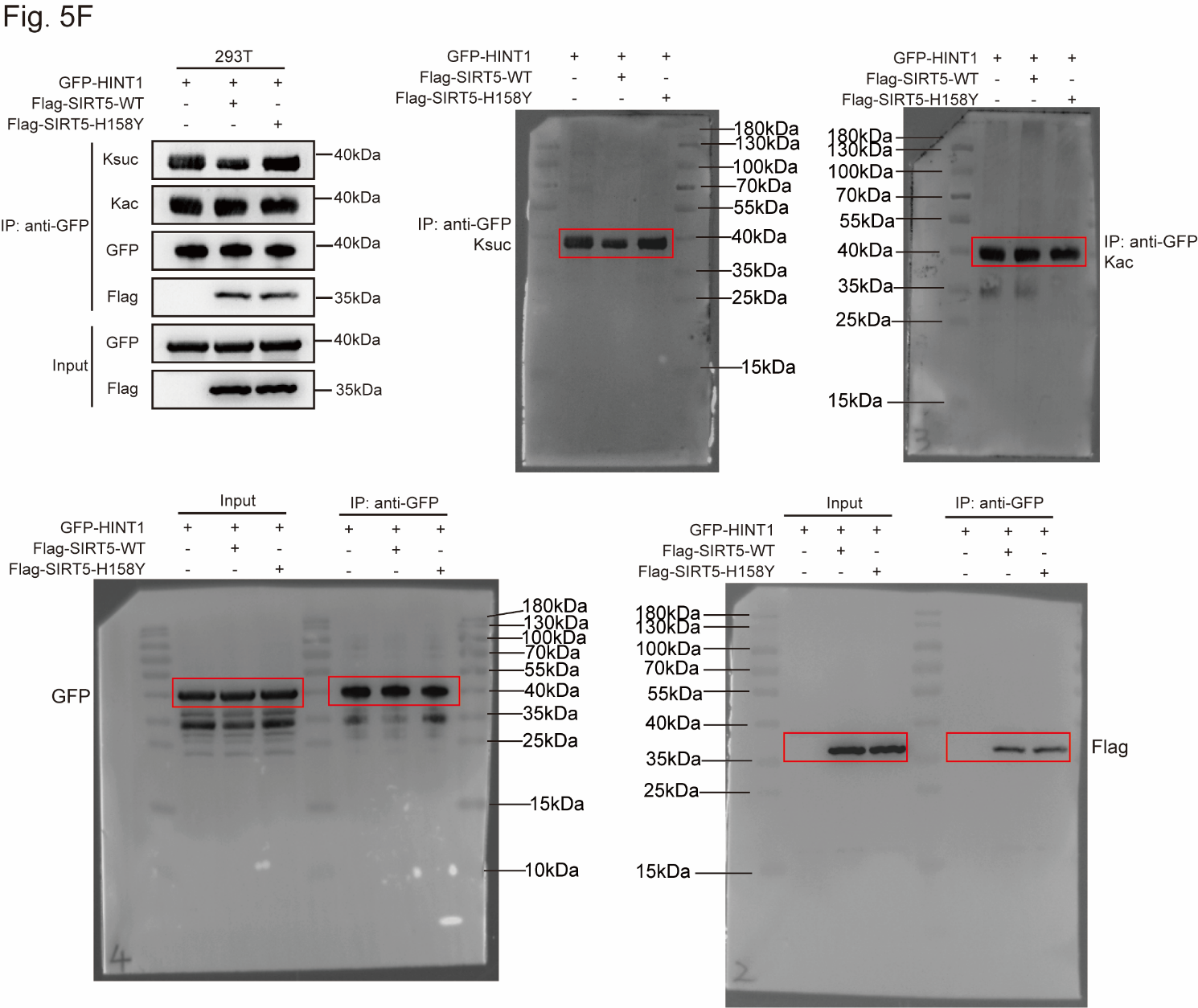


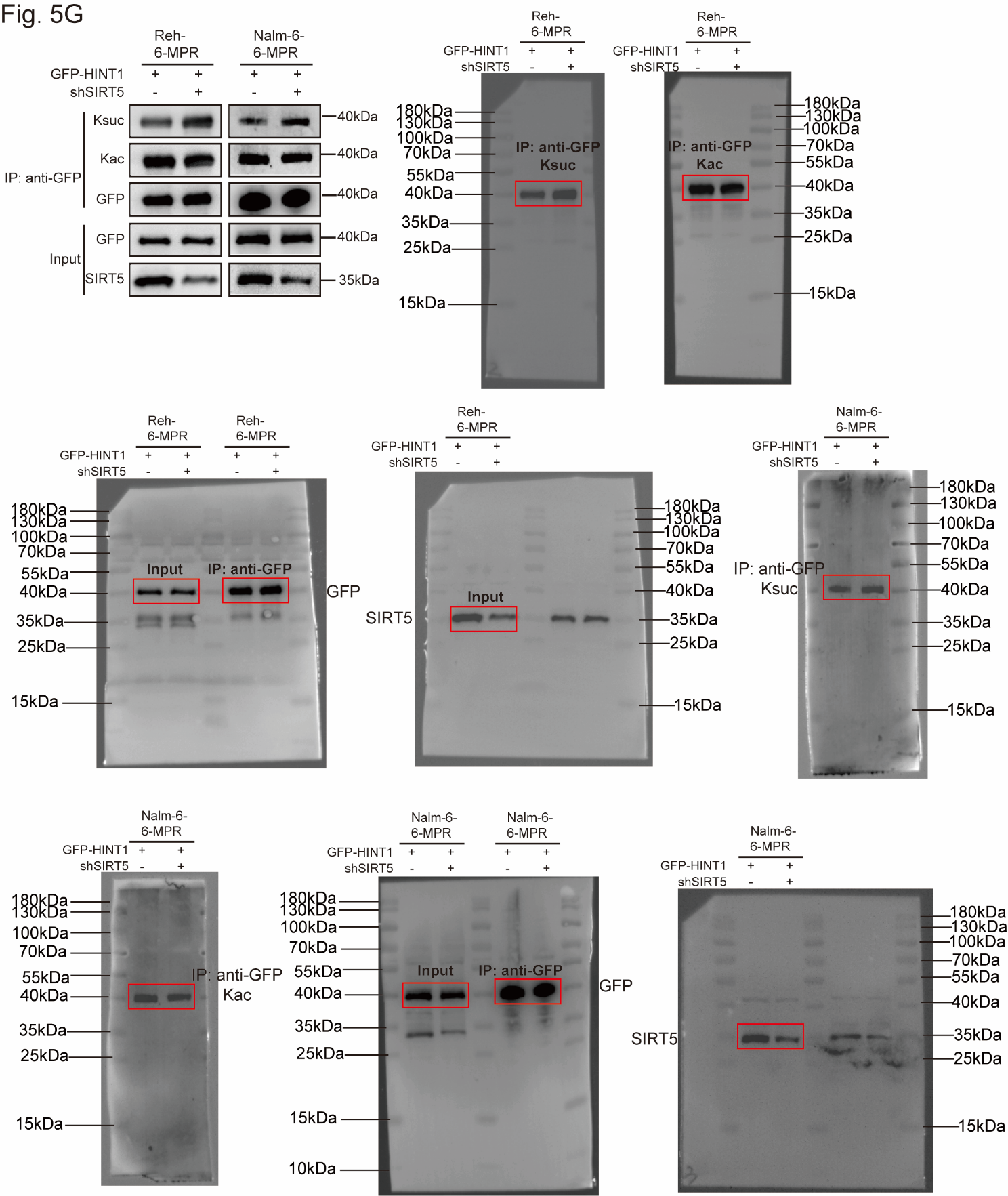


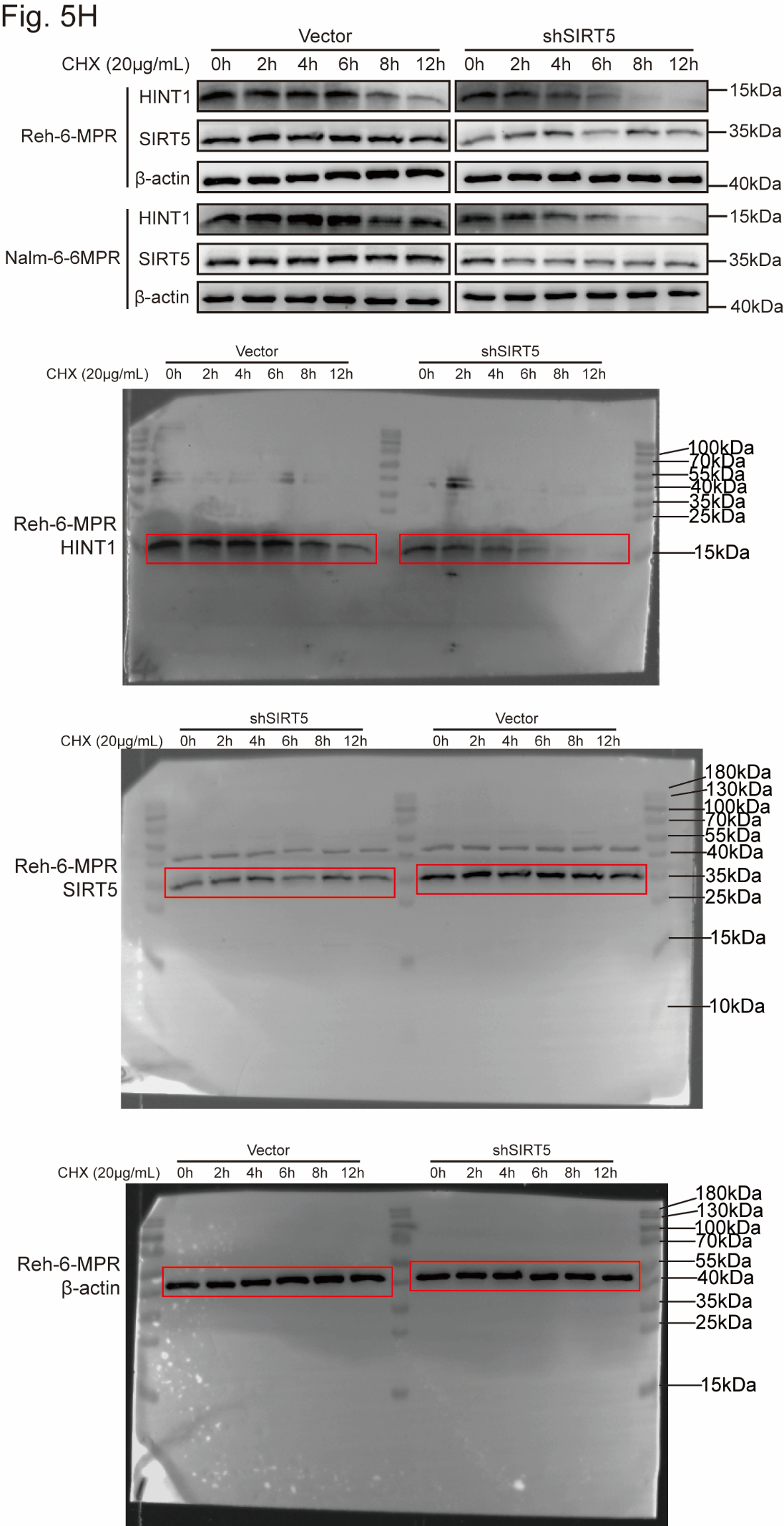

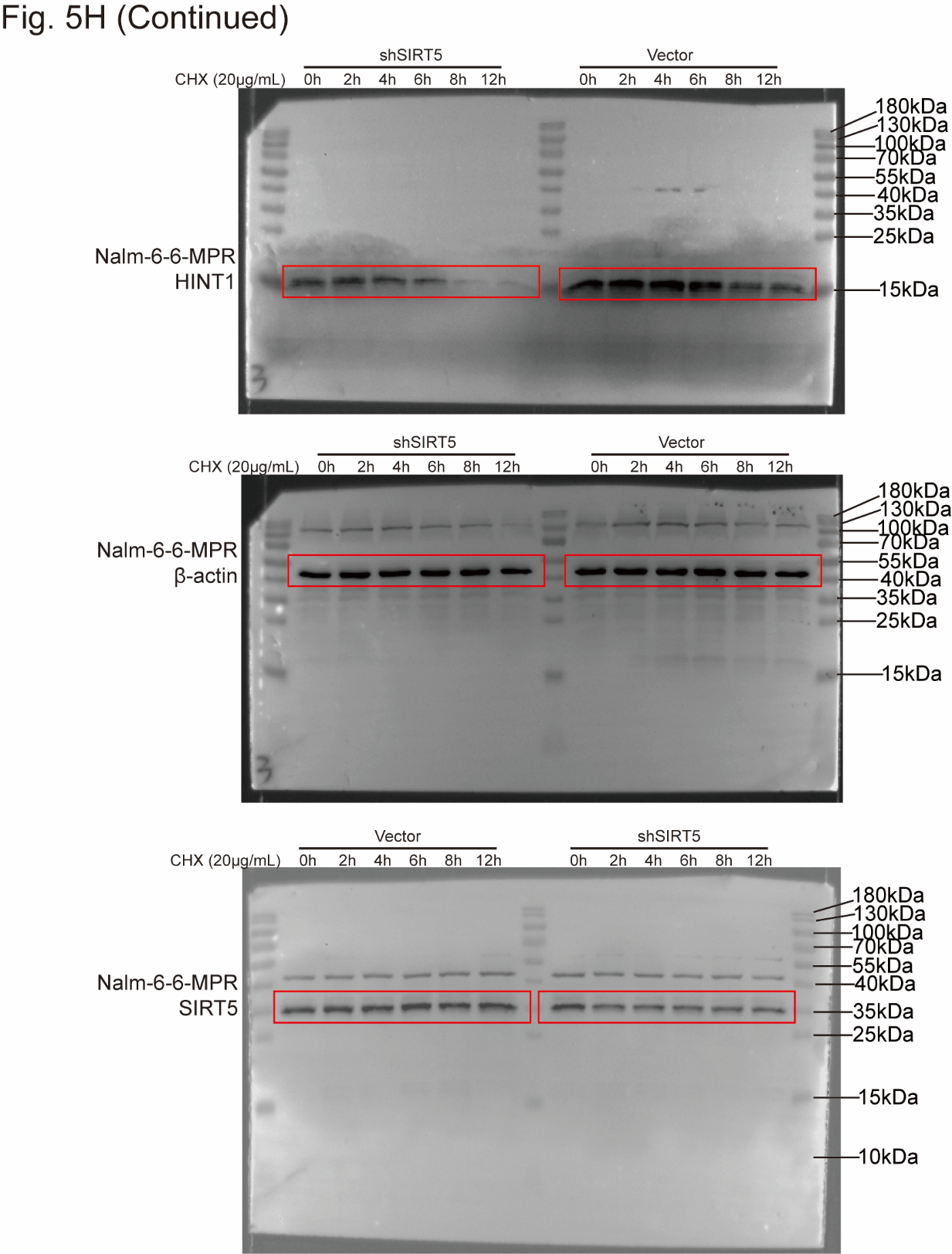


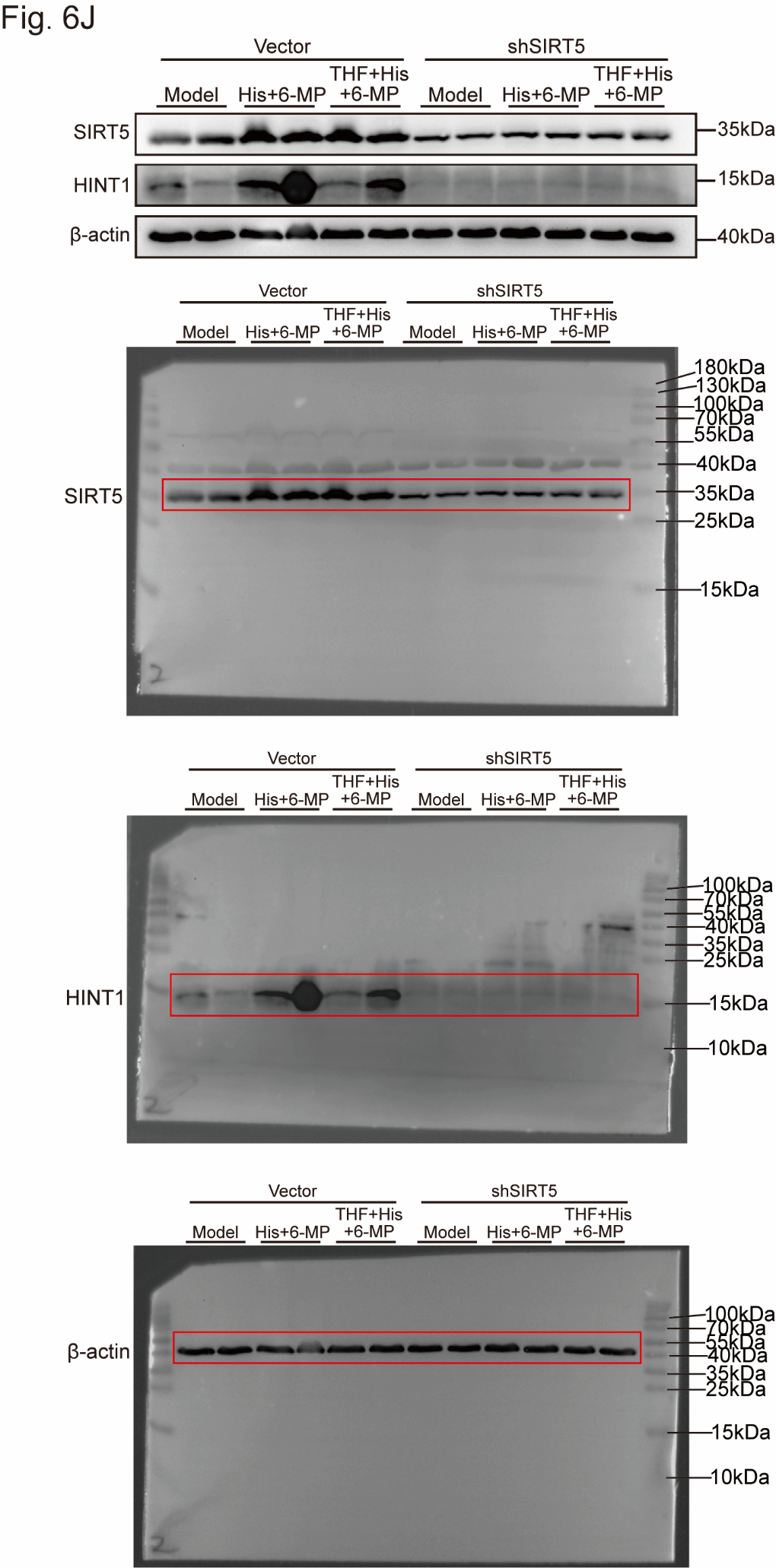


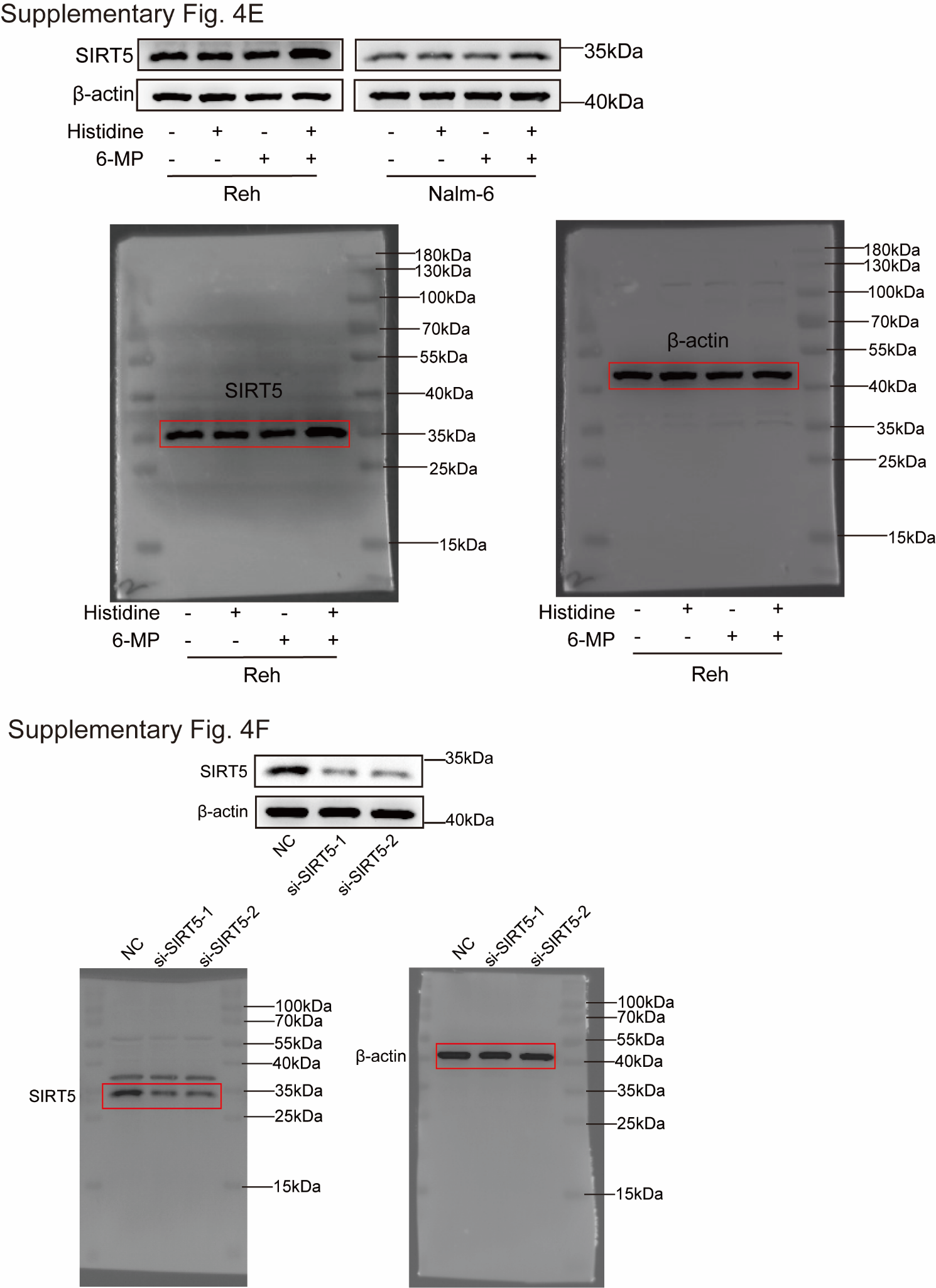


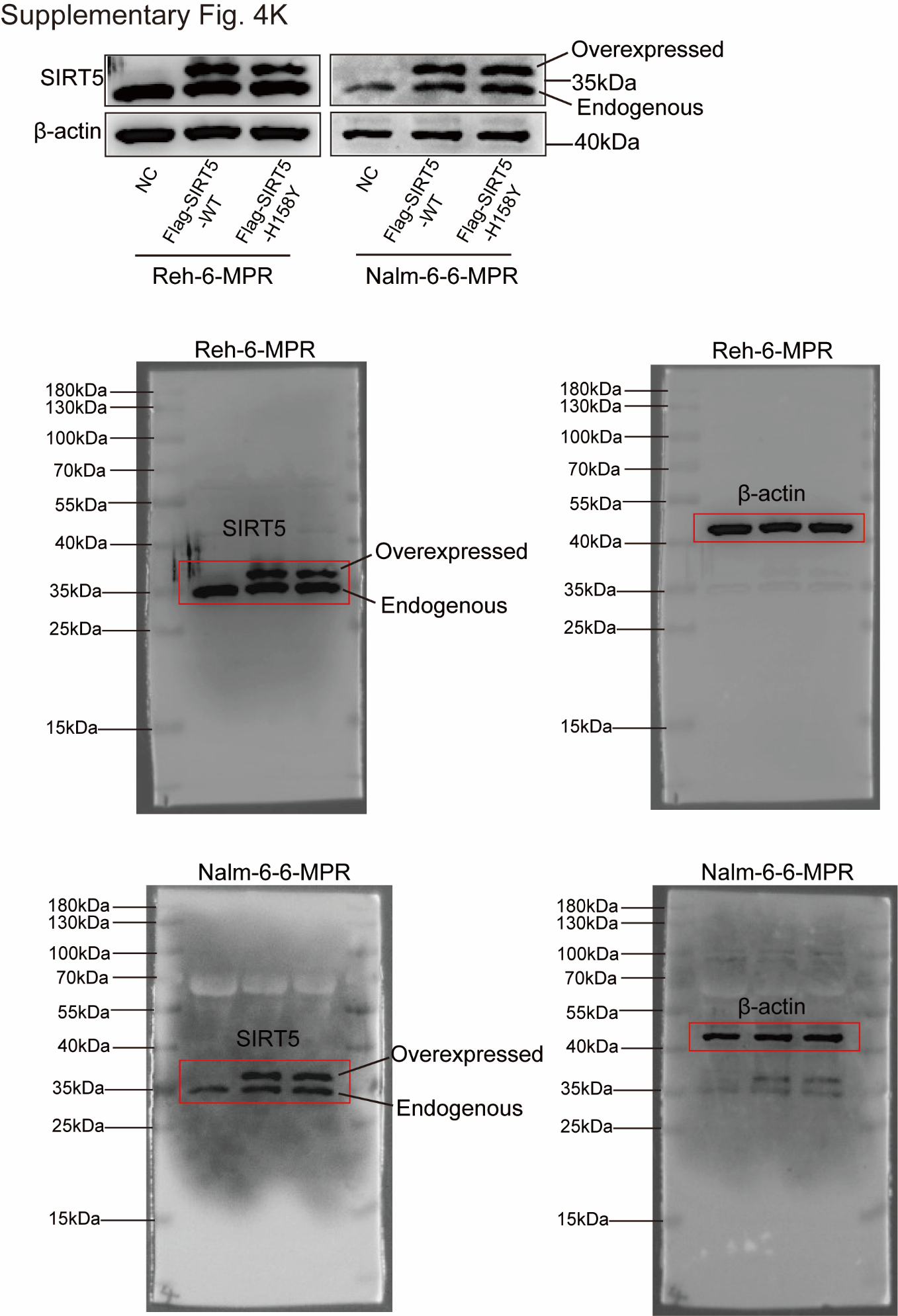

Supplement: Supplementary file 3 — Original western blots [file 41419_2024_6599_MOESM3_ESM.docx]
